# Phytochemicals from Selective Plants Have Promising Potential against SARS-CoV-2: Investigation and Corroboration through Molecular Docking, MD Simulations, and Quantum Computations

**DOI:** 10.1155/2020/6237160

**Published:** 2020-10-13

**Authors:** Kafila Kousar, Arshia Majeed, Farkhanda Yasmin, Waqar Hussain, Nouman Rasool

**Affiliations:** ^1^Department of Healthcare Biotechnology, Atta ur Rahman School of Applied Biosciences, National University of Science and Technology Islamabad, Pakistan; ^2^Medicare Health Services, Lahore, Pakistan; ^3^Department of Biotechnology, Khawaja Fareed University of Science and Technology, Rahim Yar Khan, Pakistan; ^4^National Center of Artificial Intelligence, Punjab University College of Information Technology, University of the Punjab, Lahore, Pakistan; ^5^Center for Professional and Applied Studies, Lahore, Pakistan

## Abstract

Coronaviruses have been reported previously due to their association with the severe acute respiratory syndrome (SARS). After SARS, these viruses were known to be causing Middle East respiratory syndrome (MERS) and caused 35% evanescence amid victims pursuing remedial care. Nowadays, beta coronaviruses, members of *Coronaviridae*, family order *Nidovirales*, have become subjects of great importance due to their latest pandemic originating from Wuhan, China. The virus named as human-SARS-like coronavirus-2 contains four structural as well as sixteen nonstructural proteins encoded by single-stranded ribonucleic acid of positive polarity. As there is no vaccine available to treat the infection caused by these viruses, there is a dire need for taking necessary steps against this virus. Herein, we have targeted two nonstructural proteins of SARS-CoV-2, namely, methyltransferase (nsp16) and helicase (nsp13), respectively, due to their substantial activity in viral pathogenesis. A total of 2035 compounds were analyzed for their pharmacokinetics and pharmacological properties. The screened 108 compounds were docked against both targeted proteins and were compared with previously reported known compounds. Compounds with high binding affinity were analyzed for their reactivity through DFT analysis, and binding was analyzed using molecular dynamics simulations. Through the analyses performed in this study, it is concluded that EryvarinM, Silydianin, Osajin, and Raddeanine can be considered potential inhibitors for MTase, while TomentodiplaconeB, Osajin, Sesquiterpene Glycoside, Rhamnetin, and Silydianin for helicase after these compounds are validated thoroughly using *in vitro* and *in vivo* protocols.

## 1. Introduction

Coronaviruses have become a source of multiple systematic infections in various animals [1]. These viruses have previously caused respiratory tract infections in humans, including severe acute respiratory syndrome and the Middle East respiratory syndrome [2]. As there are no specific therapeutic agents for SARS-CoV, so the use of face mask, hand washing, and careful disposal of medical equipment and other materials infected with nasal secretions are preliminary preventive measures which must be followed to avoid the spread of the pandemic virus [3]. The initial clinical manifestations in the SARS-CoV infected patients observed were fever, cough, headache, myalgia, diarrhoea, dyspnea, leucopenia, lymphopenia, thrombocytopenia, hypoxaemia, pulmonary infiltration, and disturbed hepatic and renal function leading to death in some individuals [4].

The genome of SARS-CoV is 30 kb encoding 27 proteins and has 14 ORFs. These proteins are further classified as structural proteins and nonstructural proteins. The structural proteins like nucleocapsid protein (N), pike surface glycoprotein (S), a small envelope protein (E), and matrix protein (M) are known to play a role in binding to host receptors. The genome of the virus encodes 16 nonstructural proteins like Helicase (nsp13), RNA-dependent RNA polymerase (nsp12), Papain-like protease (nsp3), main protease (nsp5) also known as 3C-like protease (3CL^pro^), and 2′O methyltransferase (nsp16). The nonstructural proteins are likely to be involved in transcription, replication, and pathogenesis and play a vital role in the life cycle of the pathogen [5–9]. Owing to the critical role of nonstructural proteins of the virus in virulence, these proteins are potential targets for antiviral drugs [9].

Helicase of coronavirus is a member of superfamily-I helicase comprising of seven conserved regions. This protein winds down partially duplex ribonucleic acid in a 5′ to 3′ way to make it open [10]. The helicase (nps13) of SARS-CoV belongs to superfamily 1 (SF1) of the six helicase superfamilies; the division is based upon various specified regions. This enzyme can open up both ribo- and deoxyribonucleic acid duplexes in 5′ to 3′ direction [11]. The associated NTPase action can target all-natural nucleotides and deoxynucleotides as substrates [12]. It is also reported that SARS-CoV-nsp12 can improve the helicase action of SARS-nsp13 by cumulative action on nucleic acid (dsRNA or dsDNA) unwinding by 2-folds [13].

There are almost a dozen conserved motifs in SF1 helicases, those involved in direct binding with nucleic acid triphosphates and nucleic acids. Two motifs known as Walker A and B are common in all of members of this super family. By investigating the structure of the enzyme, it is revealed that the two RecA-like domains (1A and 2A) constitute the catalytic site of SF1 helicases. This enzyme is known to be engaged in synthesis of viral RNA and also manipulates the DNA of host [13–15]. 2′-O-MTase (2'O methyltransferase) of coronaviruses is highly conserved and is known to play an indispensable role in viral replication and evasion from innate immunity [16]. It is also reported that the methyltransferases are vital for viral replication in cell cultures. Another protein nsp10 is also crucial for the proper functioning of MTase activity in the host cells for proliferation [17, 18]. Due to the conserved nature of this enzyme and its role in virulence, this enzyme is an ideal target for potential antiviral agents and vaccines against SARS-CoV-2, SARS-CoV, MERS-CoV, or other RNA and DNA viruses [19].

Phytochemicals have been known from ancient times for their immense potential and beneficial properties against several infectious diseases and health-related complications [20]. The phytochemicals constitute magnificent potentials, specifically antiviral properties, and can be used in treating viral infections. With time, *in silico* approaches are gaining much attention around the world for their advanced strategies and effective techniques related to the field of medical sciences. They provide an operative platform, where scientists can analyze a wide range of biological phenomena, different pathways, and molecular interactions. These methods are primarily cost-effective and consist of authentic methods which predict the results with the highest accuracy [21, 22].

The present study aims at the *in silico* analysis of two nonstructural proteins of novel coronavirus (SARS-CoV-2), i.e., helicase (nsp13) and methyltransferase (nsp16). The study is performed opting standard protocols of computer-aided drug discovery, as previously mentioned in a series of publication [23–35]. The analyses performed included ADMET analysis of phytochemicals, molecular docking of phytochemicals with targeted proteins of a novel coronavirus, binding analysis through molecular dynamics (MD) simulations, and reactivity analysis through density functional theory (DFT) calculations.

## 2. Materials and Methods

**Figure 1:**
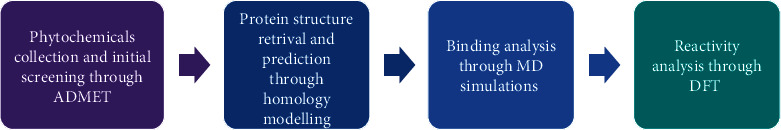
Flowchart of methodology.

Herein, the MTase and helicase of the novel coronavirus were targeted for the identification of candidate inhibitors; thus, a series of analyses were performed, and the flowchart is shown in Figure 1.

### 2.1. Obtaining the Phytochemicals

The datasets comprising of a wide range of phytochemicals (2035) were collected by using two different databases [36, 37] and also have been reported previously in [35] (Table S1). The set comprised of a variety of phytochemical groups, i.e., 322 alkaloids, 113 terpenoids, 105 aurones, 101 chalcones, 378 flavonoids, 211 lignans, 255 carboxylic acids, 301 polyphenols, and 249 quinones [35]. The 3D structures for the phytochemicals were obtained from PubChem (https://pubchem.ncbi.nlm.nih.gov/). The pharmacological and pharmacokinetic properties based on parameters of ADMET (Absorption, Distribution, Metabolism, Excretion and Toxicity) were evaluated with the help of the SwissADME web server [38] and PreADMET server [39], as reported in [23, 26, 28, 29, 31]. SwissADME server was utilized for the determination of ADME properties of the phytochemicals, while PreADMET was utilized to assess the druglikeness features and toxicity level of the drug. The structural file (.SDF) of the 2035 phytochemicals was utilized for the prediction purposes.

### 2.2. Homology Modelling

The crystal structure of MTase was available at RCSB with PDB ID: 6W61; however, helicase structure was unavailable. For protein structure prediction and homology modelling, the polypeptide sequence of SARS-CoV-2 (GenBank ID: QHD43415.1) was used, comprising 7096 residues. For mapping helicase sequence, multiple sequence alignment was performed between targeted sequence and SARS-CoV sequence (UniProt Acc ID: P0C6X7). Later on, homology modelling was performed. PSI-BLAST method was performed for template structure which was homologous to the helicase. Thus, it was observed that the sequence of helicase showed 96.08% similarity with 6YJT. The Modeller 9.18 was used to perform the homology modelling with help of 6YJT structure [40]. Opting homology modelling, 100 models were predicted and evaluated based on Discrete Optimized Protein Energy (DOPE) score. The structure quality assessment was performed by the Ramachandran Plot using RAMPAGE tool [41]. For identifying pocket, POVME 2.0 was used [42].

### 2.3. Molecular Docking and Binding Analysis

The possible inhibitory effects of these naturally occurring compounds were evaluated by the docking method against the targeted both proteins. AutoDock Tools and AutoDock Vina were used for molecular docking of proteins and ligands [43–45]. Binding energies were estimated, and interactions were analyzed. Using binding affinities, *K*_*i*_ values were also calculated by equation (1)

(1) $$\begin{array}{c} K_{i}=\frac{∆G}{e^{R\;x\;T}}, \end{array}$$

where Δ*G* represents binding energy, *T* depicts the temperature which is 298.15 k, and *R* depicts gas constant with value 1.9872036 kcal/mol. To find out the binding affinity of inhibitors with targeted proteins, docking was performed. A grid box was generated with the help of AutoDock Tools, and sizes of *x*, *y*, and *z* were determined. Grid box dimensions are provided in Table 1. Binding affinity values of these ligands were determined to evaluate how well they interact with the protein of interest. The docking was performed with 6 different exhaustiveness heuristics which were *E* = 4, *E* = 8, *E* = 16, *E* = 32, *E* = 64, and *E* = 128. However, no deviation was observed in the values of binding affinity after *E* = 8. The output files obtained from docking were used for structural analysis in the Discovery Studio 2.5 [46]; thus, 2D and 3D structural images for binding were generated.

Constant temperature MD simulations were performed to study the stability in the binding of phytochemicals with MTase and helicase using Groningen Machine for Chemical Simulations (GROMACS) v 5.0 [47]. Only those complexes were analyzed, where phytochemicals showed high binding affinity. For all those protein-ligand complexes, the optimized potential for liquid simulation (OPLS-AA) was applied, and the system was solvated with spc216 water molecules. This solvated system was neutralized by adding counter ions of Na^+^ and Cl^−^. At the next step, this system was subjected to energy minimization with the steepest descent method, keeping step limit as 50000. Later on, constant Number Volume and Temperature (NVT) and constant Number Pressure and Temperature (NPT) equilibrations were performed with 1 atm pressure and at 300 K. Explicit water molecules were also added in the phosphoserine sites, and for all simulations, standard pH of 7.0 was considered. This set of constraints was selected due to keeping the simulations similar to the human biological system. The duration for both equilibrations was 1 ns whereas the force field used in both equilibrations was Particle Mesh Ewald (PME) with a cubic interpolation implementation [48]. The hydrogen bonds were readjusted with the help of Linear Constraint Solver (LINCS) technique while performing equilibrations [49]. The final production MD simulation was performed for 50 ns, keeping the method same as equilibrations [50].

### 2.4. Reactivity Studies through DFT

Density functional theory (DFT) analysis was performed to study the reactivity of ligands with MTase and helicase. For analysis, HOMO (highest occupied molecular orbital) and LUMO (lowest unoccupied molecular orbital) energies were computed. The Δ*E* (band energy gap) calculation was performed using the expression *E*_LUMO_ − *E*_HOMO_. These descriptors are based on quantum mechanics and its computations and were performed using the program named ORCA [51]. B3LYP exchange-correlation functional was employed for calculations, which is a hybrid exchange-correlation functional. Generally, the hybrid exchange-correlation is a combination of Hartree-Fock exact exchange functional and any other density functional. However, the targeted correlation, i.e., B3LYP, is defined as:

(2) $$\begin{array}{c} E_{xc}^{\text{B}3\text{LYP}}=E_{x}^{LDA}+a_{0}\;\left(E_{x}^{\text{HF}}-E_{x}^{\text{LDA}}\right)+a_{x}\;\left(E_{x}^{\text{GGA}}-E_{x}^{\text{LDA}}\right)+E_{c}^{\text{LDA}}+a_{c}\;\left(E_{c}^{\text{GGA}}-E_{c}^{\text{LDA}}\right), \end{array}$$

where *a*_0_ = 0.20, *a*_*x*_ = 0.72, and *a*_*c*_ = 0.81. *E*_*x*_^GGA^ is the generalized gradient approximation for the Becke 88 functional while the *E*_*c*_^GGA^ reflects the correlation functional of Lee-Yang-Parr. With this hybrid functional, local density approximation is added in the form of *E*_*c*_^LDA^ [52].

## 3. Results and Discussion

Human CoVs are zoonotic pathogens, originating from animals, infecting humans leading to respiratory complications. All HCoVs provide substantial evidence of its origin from bats [53]. Phylogenetically related CoVs in bats and humans suggest host switches. SARS-CoV switched hosts from Rhinolophid bats to palm civets and human [54]. The evolutionary studies also showed that bats also host MERS-like virus suggesting bats as MERS-CoV source; infecting humans had evidence that camels might also be its likely source [55]. These viruses can transmit to humans, directly or indirectly [56].

Currently, there is no suitable vaccine available which can be used to cure infections caused by the novel coronavirus currently named as COVID-19. It is highly needed to design a drug or a vaccine to treat this virus as well as to reduce the viral deportment so that its spread could be limited. Repurposing of drugs is a remarkable approach to opt antiviral compounds as COVID-19 drug candidates [57]. Recently, a study has been reported targeting the main protease of SARS-CoV-2, using a set of phytochemicals [30]. However, herein, another set of phytochemicals has been used to target MTase and helicase.

The criteria for evaluating the compounds based on their ADMET profiles was “Violations from Lipinski's rule = Zero; Soluble = High/Very High; Absorption in Gastrointestinal (GI) tract = Efficient or Moderate; Blood-brain barrier (BBB) permeability = No; and Toxicity/carcinogenicity =0” [58]. Through the screening criteria for their ADMET properties, 108 phytochemicals out of 2035 qualified for performing the further analysis (Table S2). These selected phytochemicals were prepared for molecular docking with our targeted proteins.

### 3.1. Structure Evaluation for Helicase

**Figure 2:**
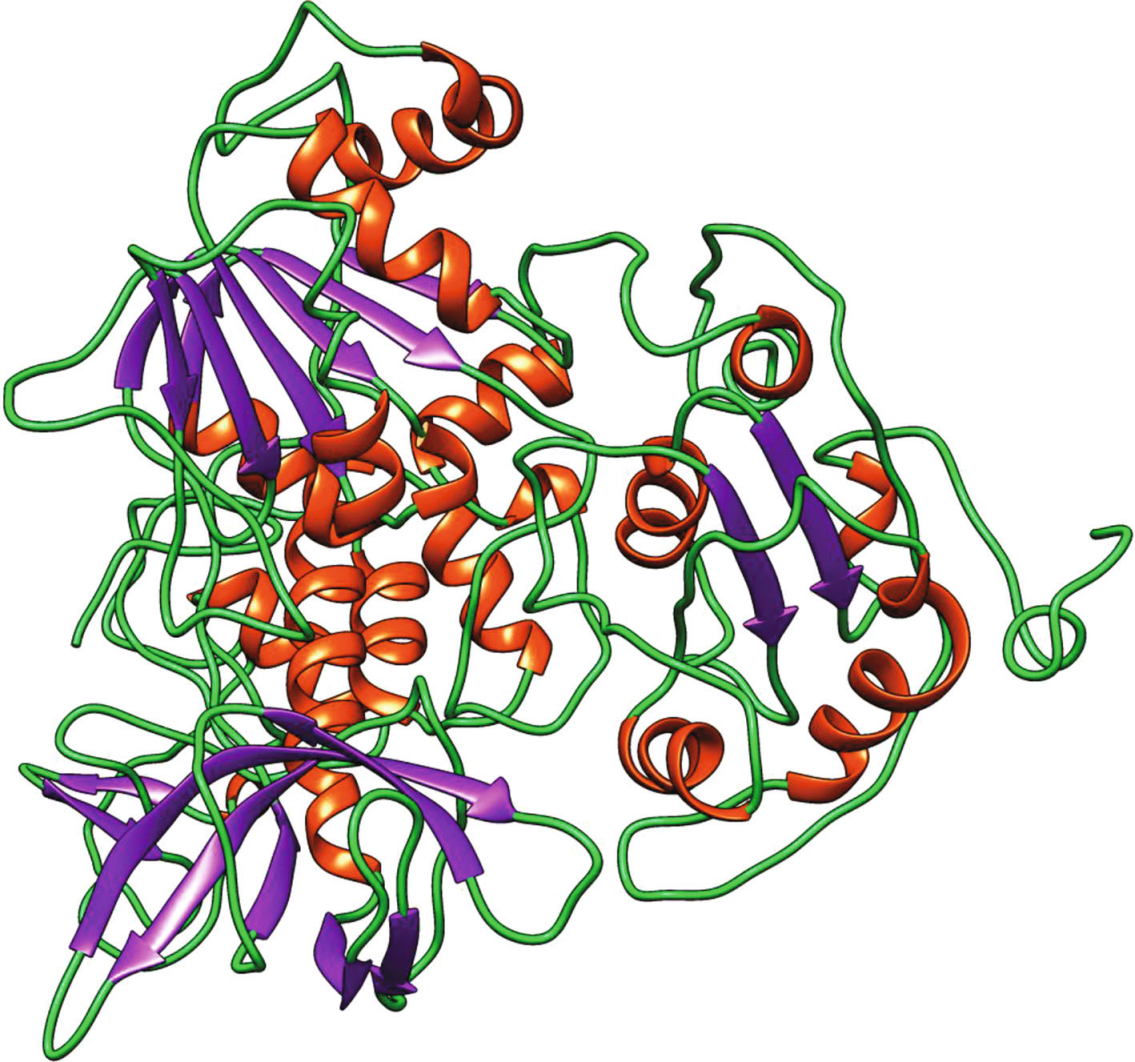
Predicted 3D model for helicase. Red depicts *α*-helices; *β*-purple depicts the strands while green depicts random coil.

The structure of helicase was modelled using homology modelling due to unavailability of X-ray crystallographic structure. As the predicted model with the lowest DOPE score was used further, it was observed to be -24913.17, and the predicted model is shown in Figure 2.

**Figure 3:**
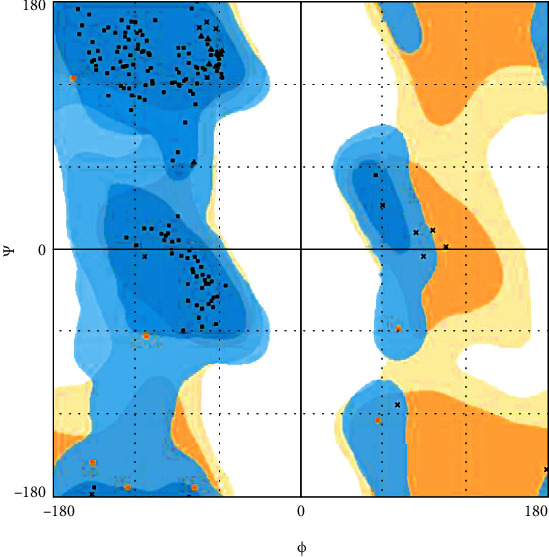
Ramachandran plot for predicted helicase model.

For quality assessment of the structure, the Ramachandran plot was generated for the predicted model using RAMPAGE tool [41]. As per the analysis of possible conformations of *φ* and *ψ* angles for individual amino acid residues in helicase model, it was observed that 98.0% residues were in the favoured region, and 1.9% residues were in the allowed region while only 0.1% residues were in outlier region (Figure 3).

### 3.2. Molecular Docking and Binding Stability

Initially, Remdesivir, Prulifloxacin, and Nelfinavir were docked with MTase and helicase, and the binding affinities of these compounds were used as a threshold for screening phytochemicals (Tables 2 and 3). Based on the results, it was observed that threshold for screening phytochemicals for MTase was -8.2 kcal/mol, and for helicase, it was -8.1 kcal/mol. Using these thresholds, phytochemicals were screened and further analyzed for stability and reactivity.

Herein, we selected Nelfinavir, Prulifloxacin, and Remdesivir from three different drug repurposing studies and docked them as controls in the present study [59, 60]. A recent paper reported an inhibitor effect of Remdesivir (a new antiviral drug) on the growth of SARS-CoV-2 *in vitro*, and an early clinical trial conducted in SARS-CoV-2 Chinese patients [61]. When Remdesivir interacted with MTase and helicase, it showed -7.0 kcal/mol and -6.8 kcal/mol binding affinity, respectively. Recent studies proposed a few drugs that target COVID-19 and suggested these compounds could be used to treat COVID-19 [62]. Prulifloxacin gave -7.6 kcal/mol with MTase and -8.1 kcal/mol with helicase. Nelfinavir, a repurposed drug, is previously known for its selectively inhibitory properties against HIV protease. This enzyme performs the posttranslational treatment of HIV propeptides. Undeveloped, noninfectious viral particles form in the cells containing this drug [63]. When Nelfinavir was docked against MTase, it showed binding affinity of -8.2 kcal/mol, while for helicase, it gave binding affinity value of -6.2 kcal/mol.

#### 3.2.1. Results of Molecular Docking of MTase of SARS-CoV-2

All 108 compounds, initially screened through ADMET, were docked with MTase of SARS-CoV-2, and the threshold used for screening phytochemicals was -8.2 kcal/mol. Thus, by applying the threshold, it was observed that only four compounds passed the threshold which were EryvarinM, Silydianin, Osajin, and Raddeanine and showed similar or better results than those of Remdesivir, Prulifloxacin, and Nelfinavir (Table 4).

**Figure 4:**
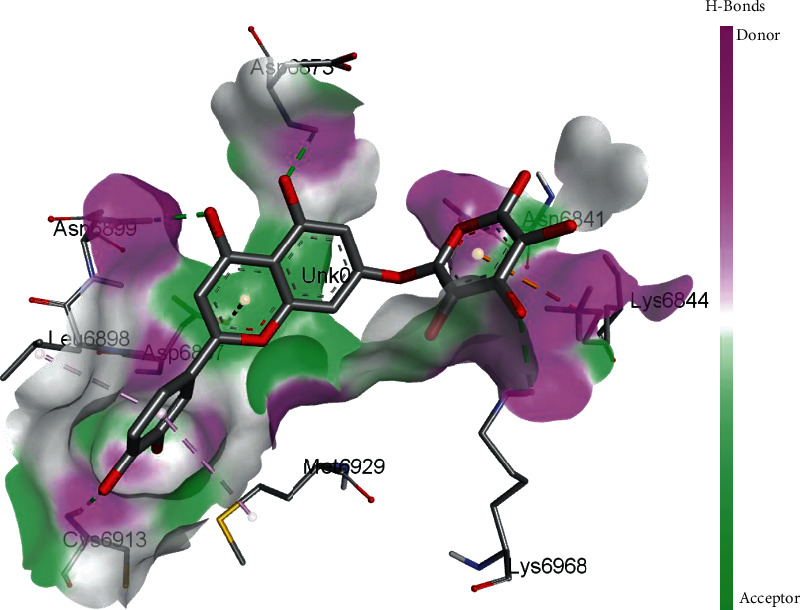
3D interaction model for EryvarinM docked with MTase.

EryvarinM docked with MTase with a binding affinity of -8.6 kcal/mol with a *K*_*i*_ value of 0.489 *μ*M while forming a conventional hydrogen bond with ASN_6841_, ASP_6873_, ASN_6899_, CYS_6913_, and LYS_6968_. Formation of pi-cation and pi-anion was observed with LYS_6844_ and ASP_6897_. Furthermore, along with these interactions, pi-alkyl interactions with MET_6929_ and LEU_6898_ were observed (Figure 4).

Silydianin docked with binding affinity -8.5 kcal/mol and *K*_*i*_ value of 0.579 *μ*M while interacting with GLY_6911_, ASP_6928_, CYS_6913_, and ASN_6899_, by forming conventional hydrogen bonds. Furthermore, it formed a carbon-hydrogen bond and pi-acceptor-acceptor interactions with MET_6929_ and ASP_6897_, while pi-alkyl and alkyl interactions with LEU_6898_, PHE_6947_, and LYS_6944_ were observed.

Osajin and Raddeanine docked with binding affinity -8.2 kcal/mol and *K*_*i*_ value of 0.961 *μ*M. Osajin formed a conventional hydrogen bond with ASP_6928_; while Raddeanine formed conventional hydrogen bonds with ASP_6912_, CYS_6913_, LEU_6898_, and ASN_6841_, however various other interactions were observed as shown in Table 4.

#### 3.2.2. Results of Molecular Docking of Helicase of SARS-CoV-2

After analyzing the ligand interactions with the binding pocket of MTase, selected phytochemicals were auto docked with helicase of SARS-CoV-2. All 108 compounds, initially screened through ADMET, were docked with helicase of SARS-CoV-2, and the threshold used for screening phytochemicals was -8.1 kcal/mol. Thus, by applying the threshold, it was observed that only five compounds passed the threshold which was TomentodiplaconeB, Osajin, Sesquiterpene Glycoside, Rhamnetin, and Silydianin and showed similar or better results than those of Remdesivir, Prulifloxacin, and Nelfinavir (Table 5).

**Figure 5:**
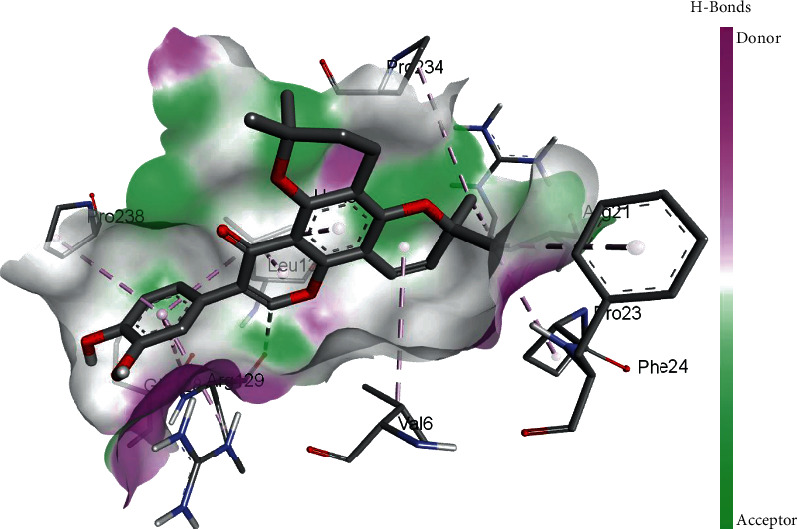
3D interaction model for TomentodiplaconeB docked with helicase.

TomentodiplaconeB formed carbon-hydrogen bond with ARG_129_, amide pi-stacked with GLU_128_, and alkyl and pi-alkyl interactions with PRO_23, 234, and 238_, LEU_132_, VAL_6_, PHE_24_, and ARG_1_. As in result, the binding affinity was -8.4 kcal/mol while *K_i_* was 0.685 *μ*M (Figure 5).

Both, Sesquiterpene Glycoside and Osajin docked with a binding affinity of -8.2 kcal/mol and *K*_*i*_ of 0.961 *μ*M. Sesquiterpene Glycoside formed conventional hydrogen bonds with ARG_21_ and ARG_129_, ASN_9_, and GLU_136_; donor interactions with ARG_22_; carbon-hydrogen bond with PHE_133_; and pi-alkyl interactions with LEU_132_, VAL_6_, and PRO_234_. Osajin formed conventional hydrogen bonds with ARG_21_ and ARG_129_ and alkyl and pi-alkyl interactions with LEU_132_ and VAL_6_.

Both, Rhamnetin and Silydianin docked with a binding affinity of -8.1 kcal/mol and *K*_*i*_ of 1.138 *μ*M. Rhamnetin formed conventional hydrogen bonds with ASP_179_, ARG_178_, and SER_310_; pi-cation and anion with ASP_534_ and ARG_560_; carbon-hydrogen bond and pi-sigma interactions with PRO_408_; and amide pi-stacked with ALA_407_ as well as pi-alkyl with PRO_406_ and ARG_409_. Silydianin formed conventional hydrogen bonds with ARG_129_ and ASN_9_. Furthermore, it formed pi-alkyl and alkyl interactions with PHE_133_, LEU_132_, VAL_6_, and PHE_24_.

#### 3.2.3. Stability in Complexes Analyzed through MD Simulations

After analyzing the interactions of ligands with targeted receptors, the MD simulations were performed to analyze the stability in complexes and binding of ligands with the proteins. These MD simulations helped in estimating the stability of binding of screened ligands with SARS-CoV-2 proteins. The radius of gyrations (*R*_*g*_) was plotted to analyze the stability in complexes, while the root means square deviation (RMSD) values were also observed for whole simulations. Average RMSD values are reported in Table 6, while graphs of the radius of gyration are shown in Figure 3.

**Figure 6:**
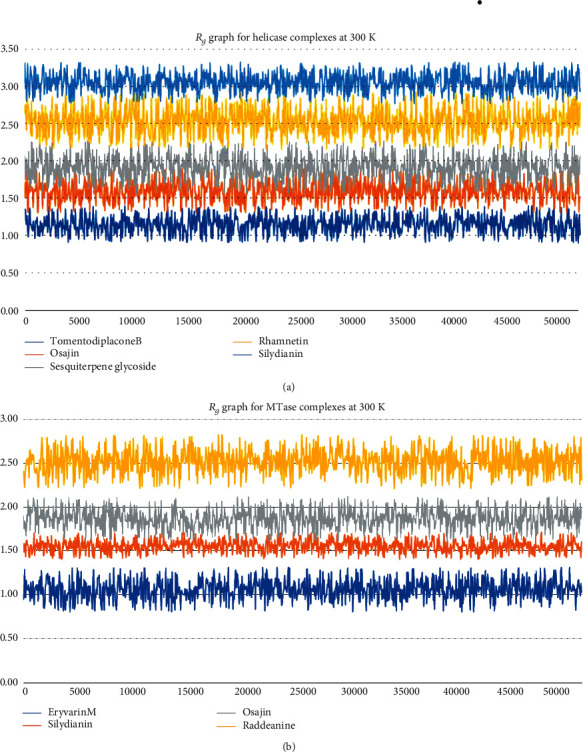
MD simulations based *R*_g_ graphs of complexes for strongly binding phytochemicals: (a) complexes of helicase; (b) complexes of MTase.

According to the results shown in Table 6, the RMSD values were observed to have mere changes, while looking in complexes of same receptors; however, these values were very low, i.e., less than 3.50 Å. Furthermore, the graphs shown in Figure 6 depicted fewer fluctuations in the radius of gyration. These values depict high stability, compactness, and stable folding of protein tertiary structure, as well as stability in protein-ligand complexes. The changes and fluctuations were observed in all complexes and not in any specific complex. The complexes with less binding affinity showed a decrease in stability and compactness, as these compounds were not bound strongly. These trends and results are also in accordance with various previously reported results [33, 60].

### 3.3. Reactivity Studies for Phytochemicals and Targeted Proteins' Complexes

Reactivity of bound phytochemicals with helicase and MTase was analyzed through density functional theory- (DFT-) based computations, which works on the principles of quantum mechanics and its descriptors. The result showed that the phytochemicals with the broad range of chemical diversity exhibited good binding interactions with both the proteins. EryvarinM, Silydianin, Osajin, and Raddeanine, which mainly exhibited best docking results for MTase, were further selected for reactivity analysis, while for helicase, TomentodiplaconeB, Osajin, Sesquiterpene Glycoside, Rhamnetin, and Silydianin were selected. For studying reactivity, band energy gap was computed using molecular orbital energy descriptors, and the results are shown in Table 7.

Lower band energy gaps show high reactivity; thus, these results exhibited in Table 7 exhibited high reactivity of phytochemicals with targeted receptors. The band energy gap values for these phytochemicals ranged from 0.112 kcal/mol to 0.133 kcal/mol and 0.116 kcal/mol to 0.128 kcal/mol, for MTase and helicase, respectively, showing narrow energy gaps and proving their high reactivity properties. B3LYP function from DFT was applied to analyze the molecular orbital energies, and it is well established in the literature that the lower band energy gap depicts higher reactivity of compounds. The reason is that band energy gaps are computed through molecular descriptors, and these descriptors are responsible for the charges transferred in a chemical reaction [64]. These energies can characterize the electrophilic or nucleophilic nature of a compound. Therefore, the screened phytochemicals illustrated the higher reactivity of these phytochemicals, as reported in various previous studies [23–25, 27, 30–33, 35, 52, 65].

## 4. Conclusion

The novel coronavirus is causing the COVID-19 worldwide, a disease which has high morbidity and a significant mortality rate. The virus, sweeping across the whole world, is pandemic as declared by the World Health Organization, and the disease was declared as a Public Health Emergency of International Concern on 30 January 2020. However, still, there exists no remedy, drug, or vaccine for the treatment of COVID-19. This study provides insights into the mechanism of selective phytochemicals, when docked against two main targets of a novel coronavirus, MTase and helicase, by showing their pharmacological properties, binding and its stability, and the reactivity. Through analysis, it is concluded that phytochemicals such as EryvarinM, Raddeanine, TomentodiplaconeB, Osajin, Sesquiterpene Glycoside, Rhamnetin, and Silydianin can be considered as candidate inhibitors for targeted proteins and as drugs, after their *in vitro* and *in vivo* examinations.

## Figures and Tables

**Table 1:** Grid box dimensions for receptors (Å^3^).

Receptor	Grid box dimensions
MTase	26 × 20 × 18
Helicase	20 × 18 × 18

**Table 2:** Results of molecular docking of Remdesivir, Prulifloxacin, and Nelfinavir with MTase of SARS-CoV-2.

Sr. no	Compound name	Binding affinity (kcal/mol)	*K*_*i*_ value (*μ*M)	Interactions with MTase of SARS-CoV-2
1	Nelfinavir	-8.2	1.138	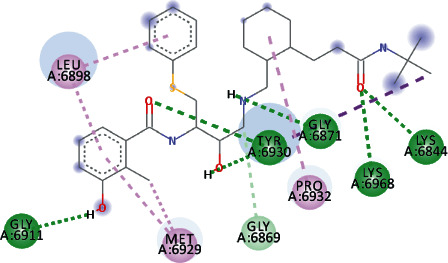
2	Prulifloxacin	-7.6	2.648	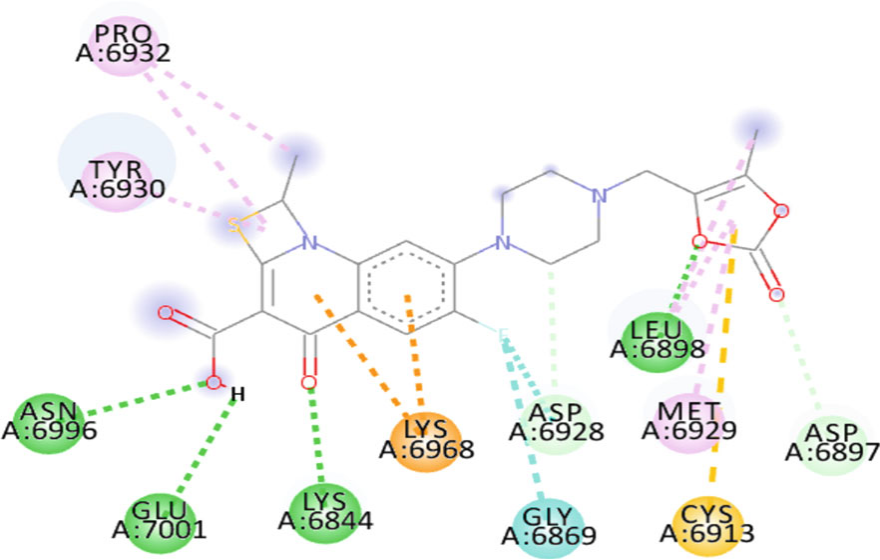
3	Remdesivir	-7.0	7.299	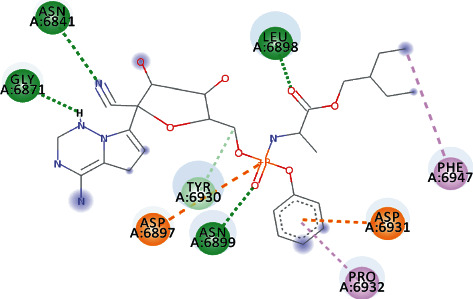

**Table 3:** Results of molecular docking of Remdesivir, Prulifloxacin, and Nelfinavir with helicase of SARS-CoV-2.

Sr. no	Compound name	Binding affinity (kcal/mol)	*K*_*i*_ value (*μ*M)	Interactions with helicase of SARS-CoV-2
1	Prulifloxacin	-8.1	1.138	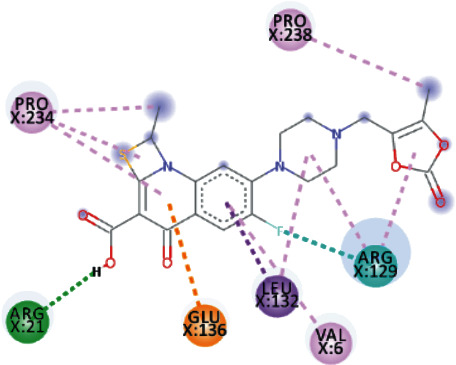
2	Remdesivir	-6.8	10.234	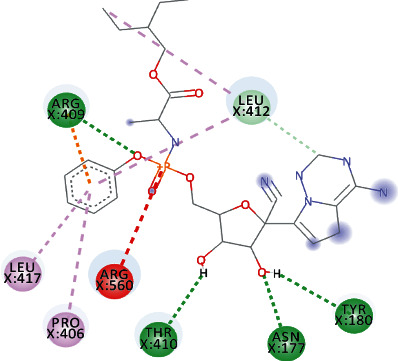
3	Nelfinavir	-6.2	28.205	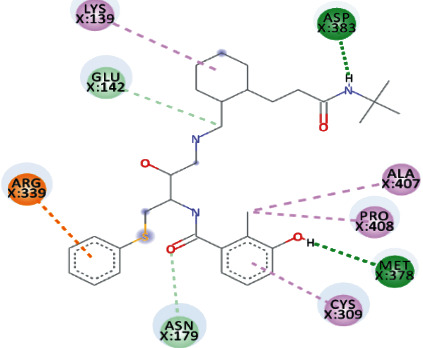

**Table 4:** Results of molecular docking of MTase of SARS-CoV-2.

Sr. no	Compound name	Binding affinity (kcal/mol)	*K*_*i*_ value (*μ*M)	Interactions with MTase of SARS-CoV-2
1	EryvarinM	-8.6	0.489	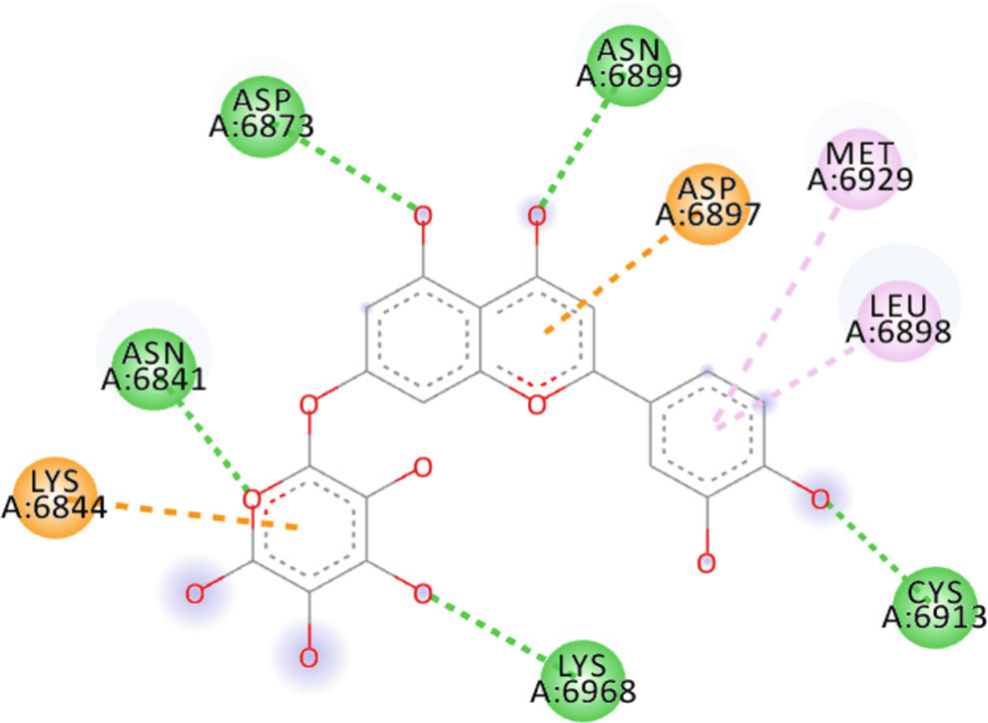
2	Silydianin	-8.5	0.579	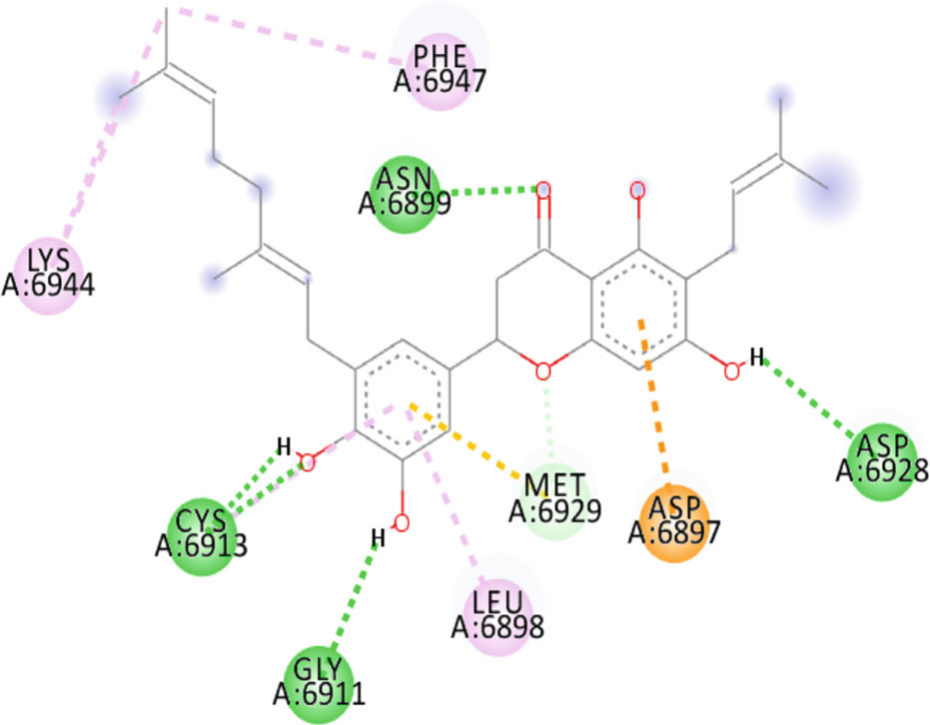
3	Osajin	-8.2	0.961	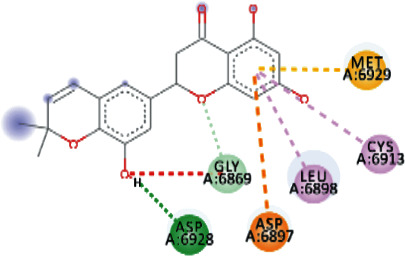
4	Raddeanine	-8.2	0.961	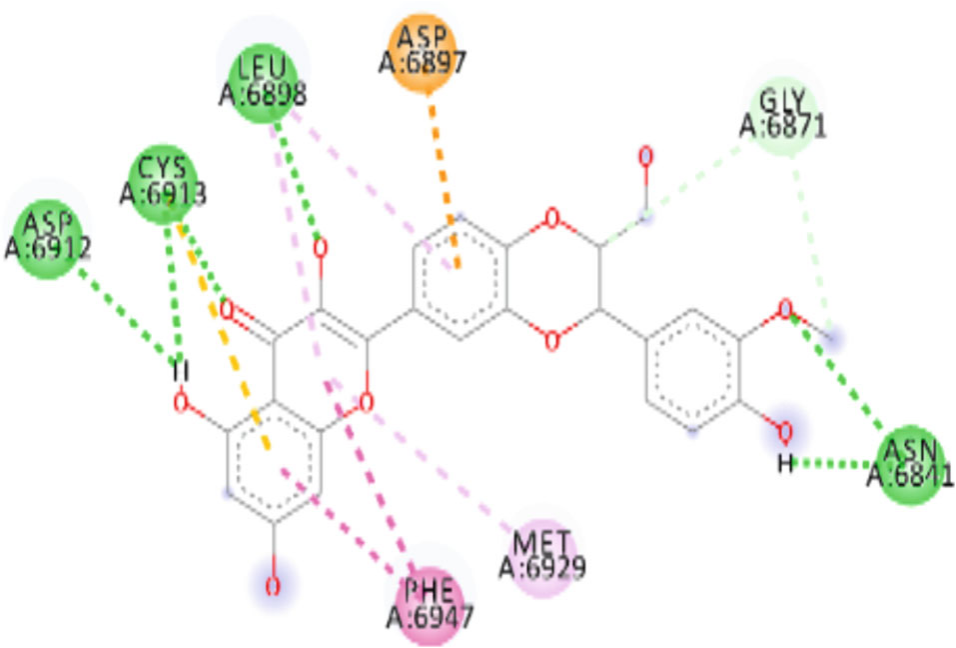

**Table 5:** Results of molecular docking of helicase of SARS-CoV-2.

Sr. no	Compound name	Binding affinity (kcal/mol)	*K*_*i*_ value (*μ*M)	Interactions with helicase of SARS-CoV-2
1	TomentodiplaconeB	-8.4	0.685	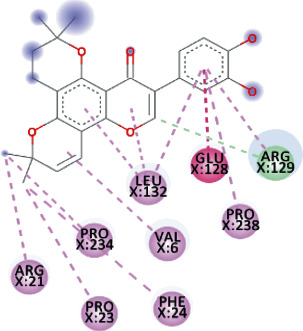
2	Osajin	-8.2	0.961	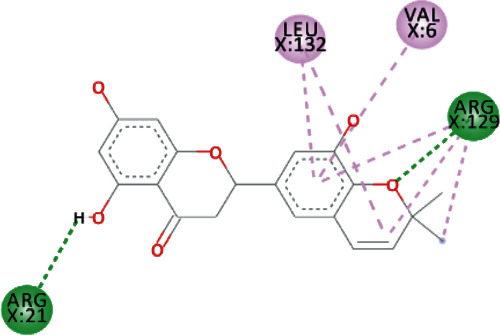
3	Sesquiterpene Glycoside	-8.2	0.961	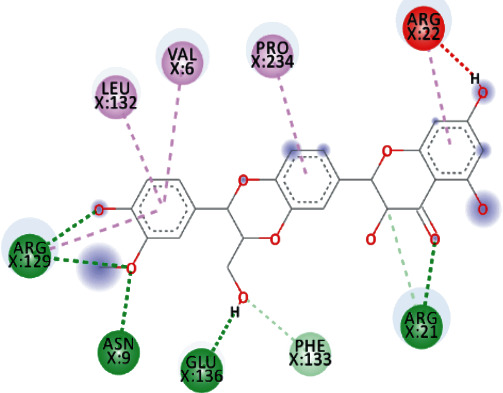
4	Rhamnetin	-8.1	1.138	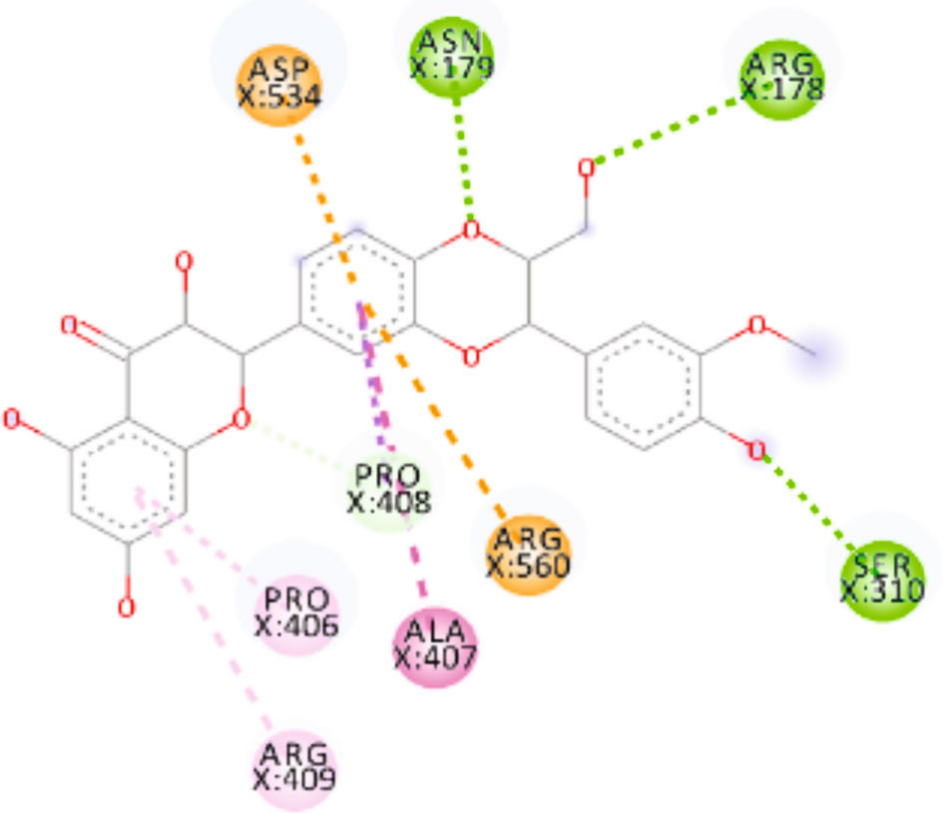
5	Silydianin	-8.1	1.138	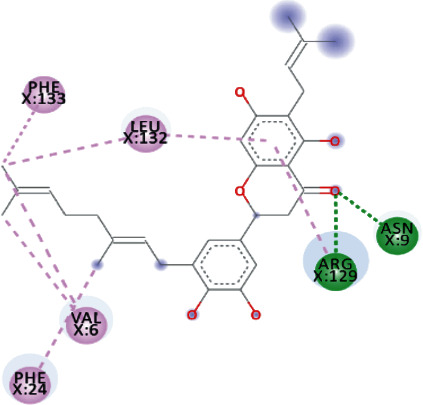

**Table 6:** Average RMSD values for all complexes.

Complex	Average RMSD (Å)
MTase-EryvarinM	1.95
MTase-Silydianin	2.05
MTase-Osajin	2.99
MTase-Raddeanine	3.12
Helicase-TomentodiplaconeB	2.11
Helicase-Osajin	2.32
Helicase-Sesquiterpene Glycoside	2.46
Helicase-Rhamnetin	2.76
Helicase-Silydianin	3.31

**Table 7:** Reactivity of phytochemicals with helicase and MTase depicted by band energy gaps.

Complexes	*E*_LUMO_ (kcal/mol)	*E*_HOMO_ (kcal/mol)	Band energy gap (Δ*E*) (kcal/mol)
MTase-EryvarinM	-0.274	-0.391	0.112
MTase-Silydianin	-0.236	-0.351	0.115
MTase-Osajin	-0.271	-0.400	0.129
MTase-Raddeanine	-0.242	-0.375	0.133
Helicase-TomentodiplaconeB	-0.280	-0.395	0.116
Helicase-Osajin	-0.218	-0.335	0.117
Helicase-Sesquiterpene Glycoside	-0.198	-0.321	0.123
Helicase-Rhamnetin	-0.303	-0.428	0.125
Helicase-Silydianin	-0.118	-0.246	0.128

## Data Availability

The data used to support the findings of this study are included within the supplementary information files.

## Supplementary Materials

Supplementary Materials Table S1: list of 2035 phytochemicals and their plants. Table S2: ADMET of Screened 108 Phytochemicals.
