# Supplementary material for: Phytochemicals from Selective Plants Have Promising Potential against SARS-CoV-2: Investigation and Corroboration through Molecular Docking, MD Simulations, and Quantum Computations
Source: Biomed Res Int. 2020 Oct 13;2020:6237160. doi: 10.1155/2020/6237160 (PMC7568149; doi:10.1155/2020/6237160)
Supplement: Supplementary Materials — Table S1: list of 2035 phytochemicals and their plants. Table S2: ADMET of Screened 108 Phytochemicals. [file 6237160.f1.docx]

**Table S1:** List of 2035 Phytochemicals and their Plants

| **Phytochemical** | **Plant Name** |
| --- | --- |
| 6-Bromohypaphorine | *Abrus precatorius* |
| Precasine | *Abrus precatorius* |
| Acalyphamide | *Acalypha indica* |
| Acalyphin | *Acalypha indica* |
| 2,4-Benzoxazolediol | *Acanthus ilicifolius* |
| 2,4,6,8-Decatetraenoic acid dehydropiperidide | *Achillea millefolium* |
| 2,4,6-Decatrienoic acid de-hydropiperidide | *Achillea millefolium* |
| 2,4-Decadienoic acid isobu-tylamide | *Achillea millefolium* |
| Homostachydrine | *Achillea millefolium* |
| N-Isobutyl-2,4,8-decatrienamide | *Achillea millefolium* |
| 8-O-Deethyl-8-O-methylkongboenine | *Aconitum chasmanthum* |
| Kongboenine | *Aconitum chasmanthum* |
| Pseudaconitine | *Aconitum deinorrhizum* |
| 3'-Methoxyacoforestinine | *Aconitum falconeri* |
| Falconerine | *Aconitum falconeri* |
| Falconerine 8-acetate | *Aconitum falconeri* |
| Faleoconitine | *Aconitum falconeri* |
| Mithaconitine | *Aconitum falconeri* |
| Pseudaconitine | *Aconitum falconeri* |
| Veratroylpseudaconine | *Aconitum falconeri* |
| Bikhaconitine | *Aconitum ferox* |
| Chasmaconitine | *Aconitum ferox* |
| Indaconitine | *Aconitum ferox* |
| Pseudaconine | *Aconitum ferox* |
| Veratroylbikhaconine | *Aconitum ferox* |
| Atidine | *Aconitum heterophyllum* |
| Dihydroatisine | *Aconitum heterophyllum* |
| Methyl N-succinoylanthranilate | *Aconitum laeve* |
| Swatinine | *Aconitum laeve* |
| 1,14-Diacetylneoline | *Aconitum napellus* |
| Aconosine | *Aconitum napellus* |
| Brachyaconitine | *Aconitum napellus* |
| Bullatine C | *Aconitum napellus* |
| Ephedrine | *Aconitum napellus* |
| Ipaconitine | *Aconitum napellus* |
| Merckonine | *Aconitum napellus* |
| Napelline | *Aconitum napellus* |
| Neoline | *Aconitum napellus* |
| Senbusine A | *Aconitum napellus* |
| Taurenine | *Aconitum napellus* |
| Pseudaconitine | *Aconitum spicatum* |
| Indaconitine | *Aconitum violaceum* |
| Kokusaginine | *Acronychia pedunculata* |
| g-Fagarine | *Aegle marmelos* |
| Marmeline | *Aegle marmelos* |
| N-4-Methoxystyrylcinnamide | *Aegle marmelos* |
| Aervolanine. | *Aerva lanata* |
| Methylaervin | *Aerva lanata* |
| (-)-Odorinol | *Aglaia odorata* |
| (+)-Odorinol | *Aglaia odorata* |
| 19-Hydroxy-3'-methoxyaglaine C | *Aglaia odorata* |
| 3',19-Dihydroxyaglaine C | *Aglaia odorata* |
| 3?-Hydroxyaglaine B | *Aglaia odorata* |
| 3'-Hydroxyaglaine C | *Aglaia odorata* |
| Aglaiastatin | *Aglaia odorata* |
| Epiodorine | *Aglaia odorata* |
| 1-Methoxycanthin-6-one | *Ailanthus altissima* |
| 1-Methoxycarbonyl-b-carboline | *Ailanthus altissima* |
| 4,5-Dihydrocanthin-6-one | *Ailanthus altissima* |
| Canthin-6-one | *Ailanthus altissima* |
| Methyl 4-methoxy-bcarboline- 1-carboxylate | *Ailanthus altissima* |
| 1-Methoxycanthin-6-one | *Ailanthus excelsa* |
| 5-Methoxycanthin-6-one | *Ailanthus excelsa* |
| Canthin-6-one | *Ailanthus excelsa* |
| Coryneine | *Alhagi pseudalhagi* |
| N-Methylmescaline | *Alhagi pseudalhagi* |
| Dihydroxytriangularine | *Alkanna tinctoria* |
| Alliarinoside | *Alliaria petiolata* |
| N-p-cis- Coumaroyltyramine | *Allium chinense* |
| Tuberosine B | *Allium tuberosum* |
| (+)-medioresinol | *Aloe Vera* |
| (+)-syringaresinol | *Aloe Vera* |
| Aloe Emodin | *Aloe Vera* |
| Srilankine | *Alseodaphne semicarpifolia* |
| 14-Ketoalstonidine | *Alstonia constricta* |
| Alstonia constricta Alkaloid | *Alstonia constricta* |
| Alstonilidine | *Alstonia constricta* |
| Alstonine | *Alstonia constricta* |
| 10-Methoxycathafoline | *Alstonia macrophylla* |
| 16-Hydroxy- Nb-demethylalstophylline oxindole | *Alstonia macrophylla* |
| 16-Hydroxyalstonal | *Alstonia macrophylla* |
| 16-Hydroxyalstonisine | *Alstonia macrophylla* |
| 6-Oxoalstophyllal | *Alstonia macrophylla* |
| 6-Oxoalstophylline | *Alstonia macrophylla* |
| Alstofoline | *Alstonia macrophylla* |
| Alstolagumine | *Alstonia macrophylla* |
| Alstomacroline | *Alstonia macrophylla* |
| Alstomacrophylline | *Alstonia macrophylla* |
| Alstomaline | *Alstonia macrophylla* |
| Alstomicine | *Alstonia macrophylla* |
| Alstonal | *Alstonia macrophylla* |
| Alstonisine | *Alstonia macrophylla* |
| Alstonoxine A | *Alstonia macrophylla* |
| Alstonoxine B | *Alstonia macrophylla* |
| Alstophyllal | *Alstonia macrophylla* |
| Alstophylline | *Alstonia macrophylla* |
| Alstoumerine | *Alstonia macrophylla* |
| Angustimalal | *Alstonia macrophylla* |
| Cathafoline N4-oxide | *Alstonia macrophylla* |
| Isoalstonisine | *Alstonia macrophylla* |
| Macralstonine | *Alstonia macrophylla* |
| Macrogentine | *Alstonia macrophylla* |
| Macrophylline$ | *Alstonia macrophylla* |
| Macrosalhine | *Alstonia macrophylla* |
| N1-Demethylalstophylline | *Alstonia macrophylla* |
| Nb-Demethylalstophylline oxindole | *Alstonia macrophylla* |
| N-Demethylalstonisine | *Alstonia macrophylla* |
| Picrinine | *Alstonia macrophylla* |
| 17- O-Acetylechitamine | *Alstonia scholaris* |
| 18(19)-Hydroxy-19,20-dihydroakuammicine | *Alstonia scholaris* |
| 19-Epischolaricine | *Alstonia scholaris* |
| 5-Epinareline ethyl ether | *Alstonia scholaris* |
| Akuammicine Nb-methosalt | *Alstonia scholaris* |
| Akuammicine Nb-oxide | *Alstonia scholaris* |
| Akuammiginone | *Alstonia scholaris* |
| Alschomine | *Alstonia scholaris* |
| Alstonamine | *Alstonia scholaris* |
| Alstoscholarine | *Alstonia scholaris* |
| Angustilobine B Nb-oxide | *Alstonia scholaris* |
| Deacetyl- 1,2-dihydro-5-oxoakuammiline | *Alstonia scholaris* |
| Echitamidine Nb-oxide 19-glucoside | *Alstonia scholaris* |
| Echitamine | *Alstonia scholaris* |
| Echitaminic acid | *Alstonia scholaris* |
| Lochneridine | *Alstonia scholaris* |
| Nb-Methylscholaricine | *Alstonia scholaris* |
| N-Demethylechitamine | *Alstonia scholaris* |
| O-Deacetyl-1,2,4,5-tetrahy-dro-2-hydroxy-1-methylakuammilinium(1+) | *Alstonia scholaris* |
| O-Ethylnareline | *Alstonia scholaris* |
| Pseudoakuammigine | *Alstonia scholaris* |
| Scholaricine | *Alstonia scholaris* |
| Scholarine | *Alstonia scholaris* |
| Echitamine | *Alstonia spectabilis* |
| Macralstonidine | *Alstonia spectabilis* |
| 11-Methoxyechitovenidine | *Alstonia venenata* |
| 11-Methoxyechitoveniline | *Alstonia venenata* |
| 19,20-Dihydropolyneuridine | *Alstonia venenata* |
| Alstolenine | *Alstonia venenata* |
| Echitovenaldine | *Alstonia venenata* |
| Echitovenidine | *Alstonia venenata* |
| Echitovenine | *Alstonia venenata* |
| Minovincinine | *Alstonia venenata* |
| Amaranthin | *Amaranthus tricolor* |
| 19-Epialstonine | *Amphicome emodi* |
| 2,4-Dodecadienoic acid isobutylamide | *Anacyclus pyrethrum* |
| Anacycline | *Anacyclus pyrethrum* |
| Dehydromatricaric acid isobutylamide | *Anacyclus pyrethrum* |
| N-(4-Hydroxyphenethyl)-2,4- decadienamide | *Anacyclus pyrethrum* |
| N-(4-Hydroxyphenethyl)-2,4-dodecadienamide | *Anacyclus pyrethrum* |
| Nmethylanacycline | *Anacyclus pyrethrum* |
| 8-Oxotetrahydropalmatine | *Anamirta cocculus* |
| N1,N4-Diferuloylputrescine | *Ananas comosus* |
| Sinapoylputrescine | *Ananas comosus* |
| Subaphylline | *Ananas comosus* |
| Ancistrocladidine | *Ancistrocladus heyneanus* |
| Ancistrocladine | *Ancistrocladus heyneanus* |
| Ancistrocladisine | *Ancistrocladus heyneanus* |
| Ancistroheynine B | *Ancistrocladus heyneanus* |
| Isoancistrocladine | *Ancistrocladus heyneanus* |
| 3,4-Dihydro-5-(4,5-dimethoxy- 2-methyl-1-naphthalenyl)-8- hydroxy-6-methoxy-1,3-dimethylisoquinoline | *Ancistrocladus tectorius* |
| 3,4-Dihydro-5-(4-hydroxy-5-methoxy-2-methyl-1-naphthalenyl)-8- hydroxy-6-methoxy-1,3-dimethylisoquinoline | *Ancistrocladus tectorius* |
| 3,4-Dihydro-6,8-dimethoxy-1,3-dimethylisoquinoline | *Ancistrocladus tectorius* |
| 6,8-Dimethoxy-1,3-dimethyl-isoquinoline, 9CI | *Ancistrocladus tectorius* |
| Ancistrocladeine | *Ancistrocladus tectorius* |
| Ancistrocladidine | *Ancistrocladus tectorius* |
| Ancistrocladine | *Ancistrocladus tectorius* |
| Ancistrotectoriline A | *Ancistrocladus tectorius* |
| Ancistrotectoriline B | *Ancistrocladus tectorius* |
| Ancistrotectorine | *Ancistrocladus tectorius* |
| 2-(2-Hydroxybutyl)piperidine | *Andrachne aspera* |
| Andrachamine | *Andrachne aspera* |
| Andrachcine | *Andrachne aspera* |
| Andrachcinidine | *Andrachne aspera* |
| Andrachcinine | *Andrachne aspera* |
| Aspertine A | *Andrachne aspera* |
| Aspertine D | *Andrachne aspera* |
| Menisdaurin | *Andrographis paniculata* |
| Noroxyhydrastinine | *Andrographis paniculata* |
| Raddeanine | *Andrographis paniculata* |
| Riboflavin | *Andrographis paniculata* |
| Thiamine | *Andrographis paniculata* |
| Uric Acid | *Andrographis paniculata* |
| Anomoline | *Annona cherimola* |
| Cherimoline | *Annona cherimola* |
| N-cis- Caffeoyltyramine | *Annona cherimola* |
| N-cis-Feruloyl-3-methoxytyramine | *Annona cherimola* |
| N-Dihydroferuloyltyramine | *Annona cherimola* |
| N-trans-Feruloyl-3-methoxytyramine | *Annona cherimola* |
| Anomurine | *Annona muricata* |
| Muricinine | *Annona muricata* |
| N,N-Dimethylanomurine | *Annona muricata* |
| Annosqualine | *Annona squamosa* |
| Benzo[c][2,7]naphthyridin-4-ol | *Annona squamosa* |
| 4-Methyl-2,6-naphthyridine | *Antirrhinum majus* |
| 2-(Malonylamino) benzoic acid | *Arachis hypogaea* |
| 2-Methylene-4-oxopentane-dioic acid | *Arachis hypogaea* |
| Argemexicaine A | *Argemone mexicana* |
| Argemexicaine B | *Argemone mexicana* |
| Argenaxine | *Argemone mexicana* |
| Dihydrochelerythrine | *Argemone mexicana* |
| Oxyhydrastinine | *Argemone mexicana* |
| Pancorine | *Argemone mexicana* |
| Lysergene | *Argyreia cuneata* |
| Festuclavine | *Argyreia nervosa* |
| Isolysergol | *Argyreia nervosa* |
| Aristolactam I | *Aristolochia indica* |
| Aristolactam Ia | *Aristolochia indica* |
| Aristolamide | *Aristolochia indica* |
| Aristolic acid | *Aristolochia indica* |
| Chondodendrine | *Aristolochia indica* |
| Aristolactam I N-glucoside | *Aristolochia longa* |
| Aristolactam Ia | *Aristolochia longa* |
| Aristolactam I | *Aristolochia rotunda* |
| Aristo red | *Aristolochia serpentaria* |
| Aristolochic acid C | *Aristolochia tagala* |
| N-Ethoxycarbonylprolinamide | *Arnica montana* |
| Xylopine | *Artabotrys suaveolens* |
| Neopellitorine B | *Artemisia dracunculus* |
| Artemisia rutifolia Alkaloid | *Artemisia rutifolia* |
| Atalaphyllidine | *Atalantia monophylla* |
| 1-Methylpyrrolidine | *Atropa belladonna* |
| Apoatropine | *Atropa belladonna* |
| Cuscohygrine | *Atropa belladonna* |
| Tropine | *Atropa belladonna* |
| Avenalumin II | *Avena sativa* |
| Avenalumin III | *Avena sativa* |
| Avenanthramide A | *Avena sativa* |
| Avenanthramide B | *Avena sativa* |
| Avenanthramide C | *Avena sativa* |
| Avenanthramide D | *Avena sativa* |
| Avenanthramide E | *Avena sativa* |
| Avenanthramide G | *Avena sativa* |
| Avenanthramide L | *Avena sativa* |
| Bisavenanthramide B2 | *Avena sativa* |
| Bisavenanthramide B3 | *Avena sativa* |
| Azcarpine | *Azima tetracantha* |
| Azimine | *Azima tetracantha* |
| Taxiphyllin | *Bambusa vulgaris* |
| Gomphrenin II | *Basella rubra* |
| Lambertine | *Berberis lamberti* |
| Oxyberberine | *Berberis lamberti* |
| Berberilycine | *Berberis lycium* |
| Chenabine | *Berberis lycium* |
| Gilgitine | *Berberis lycium* |
| Jhelumine | *Berberis lycium* |
| Karakoramine | *Berberis lycium* |
| Palmatine chloroform | *Berberis lycium* |
| Sindamine | *Berberis lycium* |
| Aromoline | *Berberis orthobotrys* |
| Pakistanine | *Berberis orthobotrys* |
| Bargustanine | *Berberis vulgaris* |
| Berberrubine | *Berberis vulgaris* |
| Betalamic acid | *Beta vulgaris* |
| Betanin | *Beta vulgaris* |
| Noracronycine | *Boenninghausenia albiflora* |
| 17-Deoxyepilamprolobine | *Bongardia chrysogonum* |
| Bongardamine | *Bongardia chrysogonum* |
| Jordanine | *Bongardia chrysogonum* |
| Brassicanal B | *Brassica campestris* |
| Cyclobrassinin | *Brassica campestris* |
| Cyclobrassinin sulfoxide | *Brassica juncea* |
| 3-Indolylmethyl glucosinolate Glucobrassicin | *Brassica napus* |
| 3-Phenylpropanoic acid | *Brassica napus* |
| Brassicanate A | *Brassica napus* |
| Isalexin | *Brassica napus* |
| 3-Indolylmethyl glucosinolate Glucobrassicin | *Brassica oleracea* |
| Brassicanal C | *Brassica oleracea* |
| Caulilexin A | *Brassica oleracea* |
| Caulilexin C | *Brassica oleracea* |
| Dioxybrassinin | *Brassica oleracea* |
| Gramine | *Brassica oleracea* |
| Isalexin | *Brassica oleracea* |
| Methoxybrassenin A | *Brassica oleracea* |
| 3-Isothiocyanato-4-methoxy-  1H-indole, 9CI | *Brassica rapa* |
| Rapalexin B | *Brassica rapa* |
| Bruceolline D | *Brucea mollis* |
| Bruceolline E | *Brucea mollis* |
| Brugine | *Bruguiera cylindrica* |
| 1,2-Hydrazinedicarboxylic acid, 9CI | *Butea monosperma* |
| 5,6-Dihydro-3,6-dioxo-1,2,4-triazine-4(3H)-carboxylic acid | *Butea monosperma* |
| Monospermin | *Butea monosperma* |
| 16- O-Acetyl-N-benzoylbuxidienine | *Buxus sempervirens* |
| 16,28-Diacetylbuxanoldine | *Buxus sempervirens* |
| 16-O-Syringoylbuxaminol E | *Buxus sempervirens* |
| 28-Acetoxy-Na-benzoylbuxidienine | *Buxus sempervirens* |
| 28-Deoxybuxanoldine | *Buxus sempervirens* |
| 30-Hydroxycyclomicrobuxene | *Buxus sempervirens* |
| 3-Benzoylcyclomicrophylline F | *Buxus sempervirens* |
| Buxadienine | *Buxus sempervirens* |
| Buxalfine | *Buxus sempervirens* |
| Buxaline C | *Buxus sempervirens* |
| Buxamine F | *Buxus sempervirens* |
| Buxamine G | *Buxus sempervirens* |
| Buxaminol B | *Buxus sempervirens* |
| Buxaminol C | *Buxus sempervirens* |
| Buxaminol E | *Buxus sempervirens* |
| Buxandonine L | *Buxus sempervirens* |
| Buxanine M | *Buxus sempervirens* |
| Buxarine | *Buxus sempervirens* |
| Buxazine | *Buxus sempervirens* |
| Buxdeltine | *Buxus sempervirens* |
| Buxene O | *Buxus sempervirens* |
| Buxepidine | *Buxus sempervirens* |
| Buxeridine C | *Buxus sempervirens* |
| Buxithienine M | *Buxus sempervirens* |
| Buxocyclamine A | *Buxus sempervirens* |
| Buxozine C | *Buxus sempervirens* |
| Buxpsiine K | *Buxus sempervirens* |
| Buxtauine M | *Buxus sempervirens* |
| Buxus Alkaloid 1 | *Buxus sempervirens* |
| Buxus Alkaloid 2 | *Buxus sempervirens* |
| Buxus Alkaloid 3 | *Buxus sempervirens* |
| Buxus Alkaloid 5 | *Buxus sempervirens* |
| Buxus Alkaloid A1 | *Buxus sempervirens* |
| Buxus Alkaloid A2 | *Buxus sempervirens* |
| Buxus Alkaloid A3 | *Buxus sempervirens* |
| Buxus Alkaloid A4 | *Buxus sempervirens* |
| Buxus Alkaloid A5 | *Buxus sempervirens* |
| Buxus Alkaloid B | *Buxus sempervirens* |
| Buxus Alkaloid B1 | *Buxus sempervirens* |
| Buxus Alkaloid B398 | *Buxus sempervirens* |
| Buxus Alkaloid B4 | *Buxus sempervirens* |
| Buxus Alkaloid B5 | *Buxus sempervirens* |
| Buxus Alkaloid B6 | *Buxus sempervirens* |
| Buxus Alkaloid B7 | *Buxus sempervirens* |
| Buxus Alkaloid B8 | *Buxus sempervirens* |
| Buxus Alkaloid BX10 | *Buxus sempervirens* |
| Buxus Alkaloid BX6 | *Buxus sempervirens* |
| Buxus Alkaloid D | *Buxus sempervirens* |
| Buxus Alkaloid M | *Buxus sempervirens* |
| Buxus Alkaloid N | *Buxus sempervirens* |
| Cyclobuxamine H | *Buxus sempervirens* |
| Cyclobuxargentine G | *Buxus sempervirens* |
| Cyclobuxine B | *Buxus sempervirens* |
| Cyclobuxine D | *Buxus sempervirens* |
| Cyclobuxophylline K | *Buxus sempervirens* |
| Cyclobuxophylline M | *Buxus sempervirens* |
| Cyclobuxophylline O | *Buxus sempervirens* |
| Cyclobuxoviridine B | *Buxus sempervirens* |
| Cyclobuxoviridine L | *Buxus sempervirens* |
| Cyclomicrobuxamine | *Buxus sempervirens* |
| Cyclomicrophylline A | *Buxus sempervirens* |
| Cyclomicrophylline C | *Buxus sempervirens* |
| Cyclomicuranine L | *Buxus sempervirens* |
| Cycloprotobuxine C | *Buxus sempervirens* |
| Cycloprotobuxine D | *Buxus sempervirens* |
| Cyclovirobuxeine A | *Buxus sempervirens* |
| Cyclovirobuxeine B | *Buxus sempervirens* |
| Cyclovirobuxine C | *Buxus sempervirens* |
| Cyclovirobuxine C | *Buxus sempervirens* |
| Cyclovirobuxine D | *Buxus sempervirens* |
| Cyclovirobuxine D | *Buxus sempervirens* |
| Cyclovirobuxine F | *Buxus sempervirens* |
| Desoxycyclobuxoxazine A | *Buxus sempervirens* |
| l-Cycloprotobuxine C | *Buxus sempervirens* |
| N20-Acetylbuxamine G | *Buxus sempervirens* |
| N20-Acetylbuxamine E | *Buxus sempervirens* |
| N3-Benzoylcycloxobuxine F | *Buxus sempervirens* |
| N-Acetylcycloprotobuxine C | *Buxus sempervirens* |
| NBenzoyl- O-acetylbuxodienine E | *Buxus sempervirens* |
| N-Benzoylcycloprotobuxine C | *Buxus sempervirens* |
| N-Benzoylcycloprotobuxoline C | *Buxus sempervirens* |
| N-Benzoylcycloprotobuxoline D | *Buxus sempervirens* |
| N-Benzoylcycloxobuxoline F | *Buxus sempervirens* |
| N-Demethylcyclomicuranine L | *Buxus sempervirens* |
| N-Methylbuxene M | *Buxus sempervirens* |
| Nor-16- acetoxybuxabenzamidienine | *Buxus sempervirens* |
| O-Tigloylcyclovirobuxeine B | *Buxus sempervirens* |
| Pseudocyclobuxine D | *Buxus sempervirens* |
| Sempervirine | *Buxus sempervirens* |
| trans-Cyclosuffrobuxinine M | *Buxus sempervirens* |
| Buxus Alkaloid M | *Buxus wallichiana* |
| Cyclovirobuxine D | *Buxus wallichiana* |
| Caesalpinine A | *Caesalpinia digyna* |
| 2-Acetylpyrrole | *Camellia sinensis* |
| (+)-Norushinsunine N-oxide | *Cananga odorata* |
| Cleistopholine | *Cananga odorata* |
| 1,18-Diamino-5,9,14-tria-zaoctadecane | *Canavalia gladiata* |
| Canavalmine | *Canavalia gladiata* |
| g-Guanidinooxypropylamine | *Canavalia gladiata* |
| 4-Hydroxyphenethylamine | *Cannabis sativa* |
| Cannabisativine | *Cannabis sativa* |
| Cannabisin A | *Cannabis sativa* |
| Cannabisin B | *Cannabis sativa* |
| Cannabisin C | *Cannabis sativa* |
| Cannabisin D | *Cannabis sativa* |
| Cannabisin E | *Cannabis sativa* |
| Cannabisin F | *Cannabis sativa* |
| Cannabisin G | *Cannabis sativa* |
| N-p-trans- Coumaroyltyramine | *Cannabis sativa* |
| N-trans-Feruloyltyramine | *Cannabis sativa* |
| Trigonelline | *Cannabis sativa* |
| Tyramine | *Cannabis sativa* |
| 15-N-Acetylcapparisine | *Capparis decidua* |
| Capparidisine | *Capparis decidua* |
| Capparisine | *Capparis decidua* |
| Capparisinine | *Capparis decidua* |
| Isocodonocarpine | *Capparis decidua* |
| N-Acetylisocodonocarpine | *Capparis decidua* |
| Capparine B | *Capparis himalayensis* |
| 5,5'''-Dicapsaicin | *Capsicum annuum* |
| Caffedymine | *Capsicum annuum* |
| Capsiamide | *Capsicum annuum* |
| Dihydrocapsaicin | *Capsicum annuum* |
| Dinorcapsaicin | *Capsicum annuum* |
| Homocapsaicin | *Capsicum annuum* |
| Homodihydrocapsaicin | *Capsicum annuum* |
| N-cis-Feruloyltyramine | *Capsicum annuum* |
| Norcapsaicin | *Capsicum annuum* |
| Nordihydrocapsaicin | *Capsicum annuum* |
| N-trans-Feruloyloctopamine | *Capsicum annuum* |
| w-Hydroxycapsaicin | *Capsicum annuum* |
| Capsaicinol | *Capsicum frutescens* |
| Hygroline | *Carallia brachiata* |
| 4-Hydroxy-3-hydroxymethyl-2-butenoic acid | *Cardiospermum halicacabum* |
| Carpaine | *Carica papaya* |
| Dehydrocarpaine I | *Carica papaya* |
| Dehydrocarpaine II | *Carica papaya* |
| Pseudocarpaine | *Carica papaya* |
| Ipobscurine A | *Carthamus tinctorius* |
| Moschamine | *Carthamus tinctorius* |
| Chaksine | *Cassia absus* |
| Isochaksine | *Cassia absus* |
| Cassiadinine | *Cassia siamea* |
| Cassiarine A | *Cassia siamea* |
| Cassiarine B | *Cassia siamea* |
| Cassyfiline | *Cassytha filiformis* |
| Cassyformine | *Cassytha filiformis* |
| Cassythic acid | *Cassytha filiformis* |
| Cassythidine | *Cassytha filiformis* |
| Cathafiline | *Cassytha filiformis* |
| O-Methylcassyformine | *Cassytha filiformis* |
| 5-(Hydroxymethyl)-1H-pyr- role-2-carboxaldehyde | *Castanea sativa* |
| 6-Epicastanospermine | *Castanospermum australe* |
| 7-Deoxy-6-epicastanospermine | *Castanospermum australe* |
| Fagomine | *Castanospermum australe* |
| (1E,3S,4S)-form Pseudomerucathine | *Catha edulis* |
| (1R,2S)-form Norephedrine | *Catha edulis* |
| Cathedulin E2 | *Catha edulis* |
| Cathedulin E3 | *Catha edulis* |
| Cathedulin E4 | *Catha edulis* |
| Cathedulin E5 | *Catha edulis* |
| Cathedulin E6 | *Catha edulis* |
| Cathedulin E8 | *Catha edulis* |
| Cathedulin K12 | *Catha edulis* |
| Cathedulin K15 | *Catha edulis* |
| Cathedulin K17 | *Catha edulis* |
| Cathedulin K19 | *Catha edulis* |
| Cathedulin K20 | *Catha edulis* |
| Cathedulin Y10 | *Catha edulis* |
| Cathedulin Y7 | *Catha edulis* |
| Cathidine B | *Catha edulis* |
| 19-Epi-3-isoajmalicine | *Catharanthus roseus* |
| 21'-Oxoleurosine | *Catharanthus roseus* |
| Ajmalicine | *Catharanthus roseus* |
| Ajmalicine hydroxyindole-nine | *Catharanthus roseus* |
| Alioline | *Catharanthus roseus* |
| Alstonine | *Catharanthus roseus* |
| Ammocalline | *Catharanthus roseus* |
| Bannucine | *Catharanthus roseus* |
| b-Carboline | *Catharanthus roseus* |
| Carosidine | *Catharanthus roseus* |
| Carosine | *Catharanthus roseus* |
| Catharanthine | *Catharanthus roseus* |
| Catharicine | *Catharanthus roseus* |
| Catharine | *Catharanthus roseus* |
| Cathindine | *Catharanthus roseus* |
| Cavincine | *Catharanthus roseus* |
| Deacetoxyleurosine | *Catharanthus roseus* |
| Ercinamine | *Catharanthus roseus* |
| Fluorocarpamine N-oxide | *Catharanthus roseus* |
| Isositsirikine | *Catharanthus roseus* |
| Leurosine | *Catharanthus roseus* |
| Neoleurocristine | *Catharanthus roseus* |
| Neoleurosidine | *Catharanthus roseus* |
| Perimivine | *Catharanthus roseus* |
| Perivine | *Catharanthus roseus* |
| Perosine | *Catharanthus roseus* |
| Pleurosine | *Catharanthus roseus* |
| Serpentine | *Catharanthus roseus* |
| Trichosetin | *Catharanthus roseus* |
| Vinleurosine, INN | *Catharanthus roseus* |
| Celogentin A | *Celosia argentea* |
| Celogentin D | *Celosia argentea* |
| Celogentin F | *Celosia argentea* |
| Celogentin G | *Celosia argentea* |
| Celogentin J | *Celosia argentea* |
| Celogentin K | *Celosia argentea* |
| Indican | *Celosia argentea* |
| Moroidin | *Celosia argentea* |
| Betalamic acid | *Celosia cristata* |
| Celosianin I | *Celosia cristata* |
| Isocelosianin I | *Celosia cristata* |
| Isocelosianin II | *Celosia cristata* |
| Chenoalbicin | *Chenopodium album* |
| N-(4- O-Methyl-trans-feruloyl)-4-O-methyldopamine | *Chenopodium album* |
| N-(4-O-Methyl-trans-caffeoyl)-3-Omethyldopamine | *Chenopodium album* |
| Chonemorphine | *Chonemorpha fragrans* |
| Biochanin C | *Cicer arietinum* |
| Javanine$ | *Cinchona calisaya* |
| 10-Methoxycinchonamine | *Cinchona ledgeriana* |
| 17-Epicinchophyllamine | *Cinchona ledgeriana* |
| 18,19-Dihydrocinchophyllamine | *Cinchona ledgeriana* |
| Aricine | *Cinchona ledgeriana* |
| Cinchonidinone | *Cinchona ledgeriana* |
| Dehydroisocinchophyllamine | *Cinchona ledgeriana* |
| Isocinchophyllamine | *Cinchona ledgeriana* |
| Tetradehydroisocinchophyllamine | *Cinchona ledgeriana* |
| Cinchonaminone | *Cinchona succirubra* |
| Cinchonidine | *Cinchona succirubra* |
| Methylsuccirubine | *Cinchona succirubra* |
| Macrocidin A | *Cirsium arvense* |
|  | *Cissampelos pareira* |
| 12-O-Methyl-(+)-curine | *Cissampelos pareira* |
| Chondodendrine | *Cissampelos pareira* |
| Cycleanine | *Cissampelos pareira* |
| Daijisong | *Cissampelos pareira* |
| Hayatine | *Cissampelos pareira* |
| Hayatinine | *Cissampelos pareira* |
| Insularine | *Cissampelos pareira* |
| Isochondodendrine | *Cissampelos pareira* |
| Menismine | *Cissampelos pareira* |
| Norimeluteine | *Cissampelos pareira* |
| 5-Methoxynoracronycine | *Citrus grandis* |
| Acrignine A | *Citrus grandis* |
| Buntanbismine | *Citrus grandis* |
| Citbismine A | *Citrus grandis* |
| Citbismine C | *Citrus grandis* |
| Citbismine E | *Citrus grandis* |
| Citracridone II | *Citrus grandis* |
| Citropone A | *Citrus grandis* |
| Prenylcitpressine | *Citrus grandis* |
| Acrimarine J | *Citrus paradisi* |
| Acrimarine N | *Citrus paradisi* |
| Azaacridone A | *Citrus paradisi* |
| Citbismine A | *Citrus paradisi* |
| Citbismine B | *Citrus paradisi* |
| Citbismine C | *Citrus paradisi* |
| Citbismine D | *Citrus paradisi* |
| Citbismine E | *Citrus paradisi* |
| Citbismine F | *Citrus paradisi* |
| Dihydroxycitracridone I | *Citrus paradisi* |
| Furoparadine | *Citrus paradisi* |
| Margrapine A | *Citrus paradisi* |
| Neoacrimarine E | *Citrus paradisi* |
| Neoacrimarine K | *Citrus paradisi* |
| 3-(3-Hydroxyphenyl)-2-pro- penoic acid, 9CI | *Citrus sinensis* |
| Citracridone I | *Citrus sinensis* |
| 1,13-Diazacyclotetracosane-2,14-dione Clausenlactam | *Clausena excavata* |
| 2-Hydroxy-3-methyl-9H-car- bazole | *Clausena excavata* |
| Clausenaquinone A | *Clausena excavata* |
| Clausevatine D | *Clausena excavata* |
| Clausevatine E | *Clausena excavata* |
| Clausevatine F | *Clausena excavata* |
| Clausevatine G | *Clausena excavata* |
| Clausine D | *Clausena excavata* |
| Clausine L | *Clausena excavata* |
| Clausine M | *Clausena excavata* |
| Clausine N | *Clausena excavata* |
| Clausine P | *Clausena excavata* |
| Clausine T | *Clausena excavata* |
| Clausine W | *Clausena excavata* |
| Furoclausine A | *Clausena excavata* |
| Mukoenine B | *Clausena excavata* |
| 1-Hydroxy-9H-carbazole-3-carboxaldehyde | *Clausena heptaphylla* |
| Clausenapin | *Clausena heptaphylla* |
| Demethylmurrayanine | *Clausena heptaphylla* |
| Heptazolidine | *Clausena heptaphylla* |
| Murrayanine | *Clausena heptaphylla* |
| Clausenamide | *Clausena lansium* |
| Homoclausenamide | *Clausena lansium* |
| Lansamide 2 | *Clausena lansium* |
| Lansimide 3$ | *Clausena lansium* |
| Lansiumamide C | *Clausena lansium* |
| Methyl 3-carbazolecarboxylate | *Clausena lansium* |
| 12'-O-Methyl-a-ergocryptine | *Claviceps purpurea* |
| 8-Hydroxy-a-ergocryptine | *Claviceps purpurea* |
| a-Ergocryptine | *Claviceps purpurea* |
| b,b-Ergoannam | *Claviceps purpurea* |
| b-Ergocryptine | *Claviceps purpurea* |
| b-Ergoptine | *Claviceps purpurea* |
| Chanoclavine I | *Claviceps purpurea* |
| Chanoclavine I aldehyde | *Claviceps purpurea* |
| Chanoclavine II | *Claviceps purpurea* |
| Cyclo(homoleucylprolyl) | *Claviceps purpurea* |
| Ergobutyrine | *Claviceps purpurea* |
| Ergocornine | *Claviceps purpurea* |
| Ergocristam | *Claviceps purpurea* |
| Ergocristine | *Claviceps purpurea* |
| Ergogaline | *Claviceps purpurea* |
| Ergoladinine | *Claviceps purpurea* |
| Ergonine | *Claviceps purpurea* |
| Ergoptine | *Claviceps purpurea* |
| Ergoptinine | *Claviceps purpurea* |
| Ergosecalinine | *Claviceps purpurea* |
| Ergosine | *Claviceps purpurea* |
| Ergosinine | *Claviceps purpurea* |
| Ergostine | *Claviceps purpurea* |
| Ergostinine | *Claviceps purpurea* |
| Ergotaminine | *Claviceps purpurea* |
| Ergovaline | *Claviceps purpurea* |
| Festuclavine | *Claviceps purpurea* |
| Isolysergic acid | *Claviceps purpurea* |
| Isolysergol | *Claviceps purpurea* |
| Lysergene | *Claviceps purpurea* |
| Lysergic acid | *Claviceps purpurea* |
| Lysergylvaline methyl ester | *Claviceps purpurea* |
| Paspalic acid | *Claviceps purpurea* |
| Pyroclavine | *Claviceps purpurea* |
| Cocsuline 2-Noxide | *Cocculus hirsutus* |
| Cohirsine | *Cocculus hirsutus* |
| Cohirsitine | *Cocculus hirsutus* |
| Cohirsitinine | *Cocculus hirsutus* |
| Corsutine | *Cocculus hirsutus* |
| Hirsutine$ | *Cocculus hirsutus* |
| Shaheenine | *Cocculus hirsutus* |
| Trilobine | *Cocculus hirsutus* |
| Coccolinine | *Cocculus laurifolius* |
| Cocculidine N-oxide | *Cocculus laurifolius* |
| Cocculidinone | *Cocculus laurifolius* |
| Cocculine | *Cocculus laurifolius* |
| Cocculitine | *Cocculus laurifolius* |
| Cocculitinine | *Cocculus laurifolius* |
| Coccuvine | *Cocculus laurifolius* |
| Coccuvinine | *Cocculus laurifolius* |
| Coclafine | *Cocculus laurifolius* |
| Coclamine | *Cocculus laurifolius* |
| Coclifoline | *Cocculus laurifolius* |
| Erythlaurine | *Cocculus laurifolius* |
| Erythramide | *Cocculus laurifolius* |
| Erythroculine | *Cocculus laurifolius* |
| Trilobine | *Cocculus laurifolius* |
| 1,2-Dehydro-2?- nortelobine | *Cocculus pendulus* |
| 1,2-Dehydroapateline | *Cocculus pendulus* |
| 1',2'-Dehydrokohatine 2b-N-oxide | *Cocculus pendulus* |
| 5'-Hydroxytelobine | *Cocculus pendulus* |
| 6'-O-Methylcocsulinine | *Cocculus pendulus* |
| Cocsiline | *Cocculus pendulus* |
| Cocsilinine | *Cocculus pendulus* |
| Cocsoline | *Cocculus pendulus* |
| Cocsuline | *Cocculus pendulus* |
| Cocsupendine | *Cocculus pendulus* |
| Kurramine 2?-a-N-oxide | *Cocculus pendulus* |
| N-Methylapateline | *Cocculus pendulus* |
| N-Norcocsulinine | *Cocculus pendulus* |
| Ophiocarpinone | *Cocculus pendulus* |
| Pendilinine | *Cocculus pendulus* |
| Pendine | *Cocculus pendulus* |
| Pendulinine | *Cocculus pendulus* |
| Siddiquamine | *Cocculus pendulus* |
| Siddiquine | *Cocculus pendulus* |
| N,N'-Diphenylurea | *Cocos nucifera* |
| Codonopsine | *Codonopsis clematidea* |
| Codonopsinine | *Codonopsis clematidea* |
| Caffeine | *Coffea arabica* |
| Scorodocarpine B | *Coffea arabica* |
| 2-Demethylcolchifoline | *Colchicum autumnale* |
| 2-Demethyldemecolcine | *Colchicum autumnale* |
| 2-O-Demethyl- g-lumicolchicine | *Colchicum autumnale* |
| 2-O-Demethyl-b-lumicolchicine | *Colchicum autumnale* |
| Allocolchicine | *Colchicum autumnale* |
| Colchiceine | *Colchicum autumnale* |
| Colchicine | *Colchicum autumnale* |
| Colchicum autumnale Alkaloid M | *Colchicum autumnale* |
| Colchifoline | *Colchicum autumnale* |
| Dithiocolchicine | *Colchicum autumnale* |
| N-Deacetyl-2-demethyl- N-formylcolchicine | *Colchicum autumnale* |
| N-Deacetyl-3-demethyl- N-formylcolchicine | *Colchicum autumnale* |
| N-Deacetyl-N-3-oxobutyrylcolchicine | *Colchicum autumnale* |
| O-Methylandrocymbine | *Colchicum autumnale* |
| Substance U | *Colchicum autumnale* |
| 3-Demethylcolchiceine | *Colchicum luteum* |
| 3-O-Demethyl- g-lumicolchicine | *Colchicum luteum* |
| Colchilutine | *Colchicum luteum* |
| Collutine | *Colchicum luteum* |
| Collutine N-oxide | *Colchicum luteum* |
| Luteicine | *Colchicum luteum* |
| Luteidine a-N-oxide | *Colchicum luteum* |
| Luteidine b-N-oxide | *Colchicum luteum* |
| Luteine$ | *Colchicum luteum* |
| Luteinine | *Colchicum luteum* |
| 1-Deoxymannonojirimycin | *Commelina communis* |
| 1-Methoxycarbonyl-b-carboline | *Commelina communis* |
| 7-O-b-DGlucopyranosyl- a-homonojirimycin. | *Commelina communis* |
| 6-Pentyl-3-piperidinol | *Conium maculatum* |
| Conhydrinone | *Conium maculatum* |
| N-Methyl-(-)-coniine | *Conium maculatum* |
| N-Methylpseudoconhydrine | *Conium maculatum* |
| Pseudoconhydrine | *Conium maculatum* |
| 14-Acetyldelosine | *Consolida ambigua* |
| Ajacine | *Consolida ambigua* |
| Ajaconine | *Consolida ambigua* |
| Delcosine | *Consolida ambigua* |
| Delphatine | *Consolida ambigua* |
| Dihydroajaconine | *Consolida ambigua* |
| Alkaloid CR1 | *Consolida regalis* |
| Mallorepine | *Conyza bonariensis* |
| Corlumine | *Corydalis govaniana* |
| Ophiocarpine | *Corydalis govaniana* |
| Dehydrocavidine | *Corydalis meifolia* |
| Corydaine | *Corydalis vaginans* |
| 8-Oxotetrahydrothalifendine | *Coscinium fenestratum* |
| 1-O-Palmitoyl-2-O-(1-Opalmitoyl-2-O-stearoyl)glycerophosphorylpseudolycorine | *Crinum asiaticum* |
| Crinisine | *Crinum asiaticum* |
| Isocraugsodin | *Crinum asiaticum* |
| Lycorine 1-Ob- D-glucoside | *Crinum asiaticum* |
| N-Demethylgalanthamine | *Crinum asiaticum* |
| O-Methylnorbelladine | *Crinum asiaticum* |
| Pratorinine | *Crinum asiaticum* |
| Pratosine | *Crinum asiaticum* |
| 1,2-Epoxyambelline | *Crinum latifolium* |
| 6-Hydroxycrinamidine | *Crinum latifolium* |
| Crinafolidine | *Crinum latifolium* |
| Crinafoline | *Crinum latifolium* |
| Latifine | *Crinum latifolium* |
| Latindine | *Crinum latifolium* |
| Latisodine | *Crinum latifolium* |
| Latisoline | *Crinum latifolium* |
| Pratorimine | *Crinum latifolium* |
| Pratorinine | *Crinum latifolium* |
| Cherylline | *Crinum powellii* |
| Crinamidine | *Crinum powellii* |
| Crinosine | *Crinum powellii* |
| Ismine | *Crinum powellii* |
| Neruscine | *Crinum powellii* |
| Powellamine | *Crinum powellii* |
| Precriwelline | *Crinum powellii* |
| Croalbidine | *Crotalaria albida* |
| Crotalarine | *Crotalaria burhia* |
| Anacrotine | *Crotalaria laburnifolia* |
| Nilgirine | *Crotalaria mucronata* |
| Crotalaria retusa Alkaloid | *Crotalaria retusa* |
| Monocrotaline | *Crotalaria spectabilis* |
| N,N'-Bis(4-aminobutyl)-1,4-butanediamine | *Crotalaria spectabilis* |
| Crotastriatine | *Crotalaria striata* |
| Crotonine | *Croton tiglium* |
| Cucubalactam | *Cucubalus baccifer* |
| Debenzoylzucchini factor B | *Cucurbita pepo* |
| N-trans-Caffeoyl-O-methyltyramine | *Cuscuta reflexa* |
| Cycasin | *Cycas circinalis* |
| Neocycasin G | *Cycas circinalis* |
| Cycasthioamide | *Cycas revoluta* |
| Neocycasin A | *Cycas revoluta* |
| Neocycasin B | *Cycas revoluta* |
| Neocycasin Ba | *Cycas revoluta* |
| Neocycasin C | *Cycas revoluta* |
| Neocycasin E | *Cycas revoluta* |
| Neocycasin J | *Cycas revoluta* |
| 14-Hydroxyantofine | *Cynanchum vincetoxicum* |
| Antofine b-N-oxide | *Cynanchum vincetoxicum* |
| 6,7-Dihydro-2,3-dimethyl-5H-cyclopentapyrazine | *Cyperus esculentus* |
| 3,13-Dihydroxylupanine | *Cytisus scoparius* |
| a-Isosparteine | *Cytisus scoparius* |
| Dopamine | *Cytisus scoparius* |
| 2',4-(N-Methylpyrrolidinyl)hygrine | *Datura innoxia* |
| 3,6-Ditigloyloxytropane | *Datura innoxia* |
| 7b-Acetoxycatuabine E | *Datura innoxia* |
| c-Tropine | *Datura innoxia* |
| 3,6-Ditigloyloxytropan-7-ol | *Datura metel* |
| Apohyoscine | *Datura metel* |
| Fastusidine | *Datura metel* |
| Fastusine | *Datura metel* |
| 2,6-Tropanediol | *Datura stramonium* |
| 6-Hydroxyhyoscyamine Noxide | *Datura stramonium* |
| Homofluorodaturatin | *Datura stramonium* |
| Physochlaine | *Datura stramonium* |
| Putrescine | *Datura stramonium* |
| 14-Aminotetradecanoic acid | *Decalepis hamiltonii* |
| Delbruline | *Delphinium brunonianum* |
| Delbrusine | *Delphinium brunonianum* |
| Dictysine | *Delphinium brunonianum* |
| Talitine B | *Delphinium caeruleum* |
| Avadharidine | *Delphinium cashmirianum* |
| Lappaconitine | *Delphinium cashmirianum* |
| Lycaconitine | *Delphinium cashmirianum* |
| Condelphine | *Delphinium denudatum* |
| Denudatidine | *Delphinium denudatum* |
| Denudatine | *Delphinium denudatum* |
| Isotalatizidine | *Delphinium denudatum* |
| Delatisine | *Delphinium elatum* |
| Deltamine | *Delphinium elatum* |
| Nordhagenine A | *Delphinium nordhagenii* |
| Nordhagenine B | *Delphinium nordhagenii* |
| Nordhagenine C | *Delphinium nordhagenii* |
| Delsemine | *Delphinium semibarbatum* |
| Delvestidine | *Delphinium vestitum* |
| Delvestine | *Delphinium vestitum* |
| 3-Hydroxy- 2-oxodendrobine | *Dendrobium nobile* |
| 4-Hydroxydendroxine | *Dendrobium nobile* |
| 6-Hydroxy-N-isopentenyldendroxine | *Dendrobium nobile* |
| Dendramine | *Dendrobium nobile* |
| Dendrobine | *Dendrobium nobile* |
| N-Isopentenyldendrobine | *Dendrobium nobile* |
| N-Isopentenyldendroxine | *Dendrobium nobile* |
| Nobiline | *Dendrobium nobile* |
| 5-Methoxy-N-methyltryptamine | *Desmodium pulchellum* |
| Bufotenine N-oxide | *Desmodium pulchellum* |
| Gramine | *Desmodium pulchellum* |
| N,N-Dimethyltryptamine N-oxide | *Desmodium pulchellum* |
| O-Methylbufotenine | *Desmodium pulchellum* |
| Desmodilactone | *Desmodium styracifolium* |
| Homoveratrylamine | *Desmodium tiliaefolium* |
| Coryneine | *Desmodium triflorum* |
| N,N-Dimethyltryptamine N-oxide | *Desmodium triflorum* |
| Duguevalline | *Desmos chinensis* |
| 2-Benzamidobenzoic acid | *Dianthus caryophyllus* |
| 4- Methoxydianthramide | *Dianthus caryophyllus* |
| 4- Methoxydianthramide M | *Dianthus caryophyllus* |
| 4-Hydroxydianthramide B | *Dianthus caryophyllus* |
| 4-Hydroxydianthramide S | *Dianthus caryophyllus* |
| 4-Methoxydianthramide B | *Dianthus caryophyllus* |
| Avenanthramide D | *Dianthus caryophyllus* |
| Dianthalexine | *Dianthus caryophyllus* |
| Dianthramide R | *Dianthus caryophyllus* |
| Dianthramine | *Dianthus caryophyllus* |
| Hydroxydianthramide R | *Dianthus caryophyllus* |
| Hydroxydianthramide S methyl ester | *Dianthus caryophyllus* |
| Chrycentrine | *Dicentra chrysantha* |
| Febrifugine | *Dichroa febrifuga* |
| Isofebrifugine | *Dichroa febrifuga* |
| Dictamnine | *Dictamnus albus* |
| Isodictamnine | *Dictamnus albus* |
| Isomaculosidine | *Dictamnus albus* |
| Dioscorine | *Dioscorea hispida* |
| Dioscorine N-oxide | *Dioscorea hispida* |
| 4-Methoxycanthin-6-one | *Drymaria cordata* |
| Drymaritine | *Drymaria diandra* |
| [(Methylamino)carbonyl]carbamic acid | *Echinops echinatus* |
| 7-Hydroxyechinozolinone | *Echinops echinatus* |
| Echinopsidine | *Echinops echinatus* |
| Echinozolinone | *Echinops echinatus* |
| Harmalan | *Elaeagnus angustifolia* |
| Nb-Methyltetrahydroharmol | *Elaeagnus angustifolia* |
| Tetrahydroharmol | *Elaeagnus angustifolia* |
| Alloelaeocarpiline | *Elaeocarpus sphaericus* |
| Elaeocarpidine | *Elaeocarpus sphaericus* |
| Elaeocarpiline | *Elaeocarpus sphaericus* |
| Elaeocarpine | *Elaeocarpus sphaericus* |
| Epialloelaeocarpiline | *Elaeocarpus sphaericus* |
| Epielaeocarpiline | *Elaeocarpus sphaericus* |
| Isoelaeocarpiline | *Elaeocarpus sphaericus* |
| Isoelaiocarpine | *Elaeocarpus sphaericus* |
| Merredissine | *emia dissecta* |
| Merresectine A | *emia dissecta* |
| Merresectine B | *emia dissecta* |
| Merresectine C | *emia dissecta* |
| Merresectine E | *emia dissecta* |
| Entadamide A | *Entada phaseoloides* |
| Entadamide B | *Entada phaseoloides* |
| Entadamide C | *Entada phaseoloides* |
| Dopamine hydrochloride | *Entada pursaetha* |
| Ephedroxane | *Ephedra intermedia* |
| N-Methylephedrine | *Ephedra sinica* |
| Camptothecin | *Ervatamia heyneana* |
| 19,20-Didehydroervatamine | *Ervatamia orientalis* |
| Dregamine | *Ervatamia orientalis* |
| Pandoline | *Ervatamia orientalis* |
| Tabernaemontanine | *Ervatamia orientalis* |
| Tabernaemontanine | *Ervatamia orientalis* |
| 11-Oxoerysopine | *Erythrina arborescens* |
| beta-Erythroidine | *Erythrina arborescens* |
| Erybidine | *Erythrina arborescens* |
| Erysodine | *Erythrina arborescens* |
| Erysodinophorine | *Erythrina arborescens* |
| Erysophorine | *Erythrina arborescens* |
| Erysopinophorine | *Erythrina arborescens* |
| Erysotramidine | *Erythrina arborescens* |
| Erysotrine | *Erythrina arborescens* |
| Erythraline | *Erythrina arborescens* |
| Erythrartine | *Erythrina arborescens* |
| Erythrascine | *Erythrina arborescens* |
| Erythratidine | *Erythrina arborescens* |
| Erythratine | *Erythrina arborescens* |
| Erythristemine | *Erythrina arborescens* |
| Isoerysopinophorine | *Erythrina arborescens* |
| Orientaline | *Erythrina arborescens* |
| Erysodine | *Erythrina fusca* |
| Erysotrine | *Erythrina fusca* |
| Erythraline | *Erythrina fusca* |
| 10,11-Dehydroerysodine | *Erythrina stricta* |
| 10,11-Dehydroerysovine | *Erythrina stricta* |
| 11-Hydroxyerysodine | *Erythrina stricta* |
| 11-Hydroxyerysovine | *Erythrina stricta* |
| Erysodine | *Erythrina stricta* |
| Erythraline | *Erythrina stricta* |
| Erythrinine | *Erythrina stricta* |
| Erysotrine | *Erythrina suberosa* |
| 2-Epierythratidine | *Erythrina variegata* |
| Decarbomethoxyerymelanthine | *Erythrina variegata* |
| Erysodienone | *Erythrina variegata* |
| Erysodine | *Erythrina variegata* |
| Erysonine | *Erythrina variegata* |
| Erysopine | *Erythrina variegata* |
| Erysopitine | *Erythrina variegata* |
| Erysotine | *Erythrina variegata* |
| Erythrartine | *Erythrina variegata* |
| Erythratidine | *Erythrina variegata* |
| Erythrinine | *Erythrina variegata* |
| Erythromotidienone | *Erythrina variegata* |
| Isococcolinine | *Erythrina variegata* |
| AbyssinoneV | *Erythrina Varigatae* |
| Erythrinins B | *Erythrina Varigatae* |
| EryvarinM | *Erythrina Varigatae* |
| EryvarinO | *Erythrina Varigatae* |
| EryvarinolsA | *Erythrina Varigatae* |
| EryvarinP | *Erythrina Varigatae* |
| EryvarinQ | *Erythrina Varigatae* |
| EryvarinR | *Erythrina Varigatae* |
| EuchrenoneB | *Erythrina Varigatae* |
| Hydroxy Erythratidine | *Erythrina Varigatae* |
| Hydroxygenistein | *Erythrina Varigatae* |
| IsoerysenegalenseinE | *Erythrina Varigatae* |
| Laburnetin | *Erythrina Varigatae* |
| Lenticin | *Erythrina Varigatae* |
| Lupiwighteone | *Erythrina Varigatae* |
| Osajin | *Erythrina Varigatae* |
| Oxyresveratrol | *Erythrina Varigatae* |
| Robustone | *Erythrina Varigatae* |
| SigmoidinA | *Erythrina Varigatae* |
| SigmoidinB | *Erythrina Varigatae* |
| SigmoidinC | *Erythrina Varigatae* |
| Stachydrine | *Erythrina Varigatae* |
| Wighteone | *Erythrina Varigatae* |
| Erythrophlamine | *Erythrophleum africanum* |
| Cinnamoylcocaine | *Erythroxylum coca* |
| Cocaine | *Erythroxylum coca* |
| Cuscohygrine | *Erythroxylum coca* |
| Dihydrocuscohygrine | *Erythroxylum coca* |
| Hygrine | *Erythroxylum coca* |
| Tropacocaine | *Erythroxylum coca* |
| 1',2-Dehydrohygrine | *Erythroxylum lucidum* |
| 3-(2-Methylbutanoyloxy)tropane-6,7-diol | *Erythroxylum monogynum* |
| Cinnamoylcocaine | *Erythroxylum monogynum* |
| Cocaine | *Erythroxylum monogynum* |
| Tropan-3a-yl 3,4,5- trimethoxybenzoate | *Erythroxylum monogynum* |
| Tropine 3,4,5-trimethoxycinnamate | *Erythroxylum monogynum* |
| Ingol | *Euphorbia antiquorum* |
| Euphococcinine | *Euphorbia atoto* |
| Ingol | *Euphorbia cornigera* |
| 2,4,6-Dodecatrienoic acid | *Euphorbia lathyris* |
| Milliamine B | *Euphorbia milii* |
| Milliamine C | *Euphorbia milii* |
| Milliamine D | *Euphorbia milii* |
| Milliamine E | *Euphorbia milii* |
| Milliamine F | *Euphorbia milii* |
| Milliamine G | *Euphorbia milii* |
| Milliamine I | *Euphorbia milii* |
| Milliamine J | *Euphorbia milii* |
| Milliamine L | *Euphorbia milii* |
| Milliamine N | *Euphorbia milii* |
| Ingol | *Euphorbia nivulia* |
| Ingol | *Euphorbia resinifera* |
| Ingol | *Euphorbia tirucalli* |
| Tirucalicine | *Euphorbia tirucalli* |
| 7-Methoxy-b-carboline-1- propanoic acid | *Eurycoma longifolia* |
| Pentyl b-carboline-1-propionate | *Eurycoma longifolia* |
| Evolitrine | *Evodia lunu-ankenda* |
| Evomeliaefolin | *Evodia meliaefolia* |
| 1- Methyl-2-(4,7-tridecadienyl)-4(1H)- quinolinone | *Evodia rutaecarpa* |
| 1-Methyl- 2-(10-pentadecenyl)-4(1H)-quinolinone | *Evodia rutaecarpa* |
| 1-Methyl- 2-(9-pentadecenyl)-4(1H)-quinolinone | *Evodia rutaecarpa* |
| 1-Methyl-2- (5-undecenyl)-4(1H)-quinolinone | *Evodia rutaecarpa* |
| 1-Methyl-2-nonyl-4(1H)-quinolinone | *Evodia rutaecarpa* |
| 1-Methyl-2-undecyl-4(1H)-quinolinone, 9CI | *Evodia rutaecarpa* |
| 2-(10-Oxoundecyl)-4(1H)-quinolinone | *Evodia rutaecarpa* |
| 2-Dodecyl-4-hydroxyquino-line | *Evodia rutaecarpa* |
| 4-Hydroxy-2-pentylquinoline | *Evodia rutaecarpa* |
| 7-Carboxyevodiamine | *Evodia rutaecarpa* |
| Acetonylevodiamine | *Evodia rutaecarpa* |
| Dihydroevocarpine | *Evodia rutaecarpa* |
| Evodiamine | *Evodia rutaecarpa* |
| Evodiaxinine | *Evodia rutaecarpa* |
| Fabianine | *Fabiana imbricata* |
| Fagopyrine | *Fagopyrum esculentum* |
| Berberrubine | *Fibraurea chloroleuca* |
| Dehydrodiscretine | *Fibraurea chloroleuca* |
| Palmatrubine | *Fibraurea chloroleuca* |
| Fibranine | *Fibraurea tinctoria* |
| Communesin B | *Ficus microcarpa* |
| 2-Ethyl-4-methylthiazole | *Foeniculum vulgare* |
| Cevanine | *Fritillaria imperialis* |
| Forticine | *Fritillaria imperialis* |
| Fritillaria imperialis Alkaloid R | *Fritillaria imperialis* |
| Harepermine | *Fritillaria imperialis* |
| Impericine | *Fritillaria imperialis* |
| Imperoline | *Fritillaria imperialis* |
| Isobaimonidine | *Fritillaria imperialis* |
| 12S-hydroxyandrographolide | *Fumaria Indica* |
| 19-hydroxy-8 (17), 13-labdadien-15, 16-olide | *Fumaria Indica* |
| 3-oxo-14-deoxy-andrographolide | *Fumaria Indica* |
| Ascorbic Acid | *Fumaria Indica* |
| Dihydrocoptisine | *Fumaria indica* |
| Epoxy | *Fumaria Indica* |
| Estafin | *Fumaria Indica* |
| Feruloyltyramine | *Fumaria Indica* |
| Fumaritine N-oxide | *Fumaria Indica* |
| Fumarizine | *Fumaria indica* |
| Hydroxyanhydro | *Fumaria Indica* |
| Isoach | *Fumaria Indica* |
| Narceimine | *Fumaria indica* |
| Narlumicine | *Fumaria indica* |
| N-p-trans- Coumaroyltyramine | *Fumaria indica* |
| Pantothenic Acid | *Fumaria Indica* |
| Papracinine | *Fumaria indica* |
| Papracinine | *Fumaria Indica* |
| Paprafumine | *Fumaria indica* |
| Paprafumine | *Fumaria Indica* |
| Papraine | *Fumaria indica* |
| Papraine | *Fumaria Indica* |
| Paprarine | *Fumaria Indica* |
| Dihydrofumariline 1 | *Fumaria officinalis* |
| Fumaric acid | *Fumaria officinalis* |
| Fumariline | *Fumaria officinalis* |
| Fumaritine | *Fumaria officinalis* |
| Fumarofine | *Fumaria officinalis* |
| N-Methyloxohydrasteine | *Fumaria officinalis* |
| O-Methylfumarophycine | *Fumaria officinalis* |
| Parfumidine | *Fumaria officinalis* |
| Capnoidine | *Fumaria vaillantii* |
| Egenine | *Fumaria vaillantii* |
| Fumaramine | *Fumaria vaillantii* |
| Fumariline | *Fumaria vaillantii* |
| Fumvailine | *Fumaria vaillantii* |
| Hydrastidine | *Fumaria vaillantii* |
| Ledecorine | *Fumaria vaillantii* |
| N-Methyladlumine | *Fumaria vaillantii* |
| Norjuziphine | *Fumaria vaillantii* |
| Norpallidine | *Fumaria vaillantii* |
| Parfumidine | *Fumaria vaillantii* |
| Parfumine | *Fumaria vaillantii* |
| Vaillantine | *Fumaria vaillantii* |
| (4-Hydroxy-3-methyl-2-bute-nyl)guanidine | *Galega officinalis* |
| Galegine | *Galega officinalis* |
| N-(3-Methyl-2-butenyl)guanidine | *Galega officinalis* |
| Gaultherialine A | *Gaultheria nummularioides* |
| (Z)-Akuammidine | *Gelsemium elegans* |
| 11-Hydroxyhumantenine | *Gelsemium elegans* |
| 11-Methoxygelsemamide | *Gelsemium elegans* |
| 11-Methoxyhumantenine | *Gelsemium elegans* |
| 14,15-Dihydroxygelsenicine | *Gelsemium elegans* |
| 14-Acetoxygelselegine | *Gelsemium elegans* |
| 14-Acetoxygelsenicine | *Gelsemium elegans* |
| 14-Hydroxy-19-oxogelsenicine | *Gelsemium elegans* |
| 14-Hydroxyelegansamine | *Gelsemium elegans* |
| 14-Hydroxygelsenicine | *Gelsemium elegans* |
| 19a-Hydroxygelsamydine | *Gelsemium elegans* |
| 19R-Hydroxy-11-methoxygelselegine | *Gelsemium elegans* |
| 19R-Hydroxydihydrogelsevirine | *Gelsemium elegans* |
| 19R-Kouminol | *Gelsemium elegans* |
| 19S-Kouminol | *Gelsemium elegans* |
| 20-Hydroxydihydrorankinidine | *Gelsemium elegans* |
| Gelsamydine | *Gelsemium elegans* |
| Gelsebanine | *Gelsemium elegans* |
| Gelsedilam | *Gelsemium elegans* |
| Gelsefuranidine | *Gelsemium elegans* |
| Gelseiridone | *Gelsemium elegans* |
| Gelsemine | *Gelsemium elegans* |
| Gelsemine N-oxide | *Gelsemium elegans* |
| Gelsenicine | *Gelsemium elegans* |
| Gelsevirine | *Gelsemium elegans* |
| Humantenine | *Gelsemium elegans* |
| Hydrakouminol | *Gelsemium elegans* |
| Koumicine | *Gelsemium elegans* |
| Koumine | *Gelsemium elegans* |
| Oliveramine | *Gentiana olivieri* |
| 1,2-Didemethylcolchicine | *Gloriosa superba* |
| 2,3-Didemethylcolchicine | *Gloriosa superba* |
| 2-Demethylcolchicine | *Gloriosa superba* |
| 2-O-Demethyl- g-lumicolchicine | *Gloriosa superba* |
| 2-O-Demethyl-b-lumicolchicine | *Gloriosa superba* |
| Colchicine | *Gloriosa superba* |
| Colchicoside | *Gloriosa superba* |
| N-Deacetyl- N-formyl-2-O-demethyl-b-lumicolchicine | *Gloriosa superba* |
| NDeacetyl- N-formyl-g-lumicolchicine | *Gloriosa superba* |
| N-Deacetyl-2,3-didemethylcolchicine | *Gloriosa superba* |
| N6-Methylagmatine | *Glycine max* |
| Piperidine | *Glycine max* |
| 2,4-Dihydroxy-3-phenyl-quinoline | *Glycosmis arborea* |
| Arboricine$ | *Glycosmis arborea* |
| Glycoborinine | *Glycosmis arborea* |
| Glycoamide A | *Glycosmis cochinchinensis* |
| Glycoamide B | *Glycosmis cochinchinensis* |
| Eustifoline A | *Glycosmis mauritiana* |
| Glycozolidine | *Glycosmis mauritiana* |
| Illukumbin B | *Glycosmis mauritiana* |
| Methylillukumbin A | *Glycosmis mauritiana* |
| 3-Methyl-9H-carbazole | *Glycosmis pentaphylla* |
| Carbalexin C | *Glycosmis pentaphylla* |
| Carbazole | *Glycosmis pentaphylla* |
| De-N-methylacronycine | *Glycosmis pentaphylla* |
| De-N-methylnoracronycine | *Glycosmis pentaphylla* |
| Glycosinine | *Glycosmis pentaphylla* |
| Glycozolidol | *Glycosmis pentaphylla* |
| Glycozoline | *Glycosmis pentaphylla* |
| Licorice glycoside E | *Glycyrrhiza uralensis* |
| Gomphrenin I | *Gomphrena globosa* |
| Gomphrenin III | *Gomphrena globosa* |
| Isogomphrenin I | *Gomphrena globosa* |
| Isogomphrenin II | *Gomphrena globosa* |
| N1,N4-Diferuloylputrescine | *Gomphrena globosa* |
| Acetopine | *Gossypium hirsutum* |
| Phaeanthine | *Gyrocarpus americanus* |
| 3-O-Acetylsanguinine | *Haemanthus multiflorus* |
| Isogambirdine | *Hamelia patens* |
| Buchapine | *Haplophyllum tuberculatum* |
| Haplotubine | *Haplophyllum tuberculatum* |
| Emetine | *Hedera helix* |
| Cyclocapitelline | *Hedyotis capitellata* |
| Isochrysotricine | *Hedyotis capitellata* |
| Isocyclocapitelline | *Hedyotis capitellata* |
| N1,N4-Dicaffeoylputrescine | *Helianthus annuus* |
| N1,N4-Di-p-coumaroylputrescine | *Helianthus annuus* |
| Coromandalinine | *Heliotropium curassavicum* |
| Curassanecine | *Heliotropium curassavicum* |
| Curassavine | *Heliotropium curassavicum* |
| Curassavine N-oxide | *Heliotropium curassavicum* |
| Curassavinine | *Heliotropium curassavicum* |
| Heliovinine | *Heliotropium curassavicum* |
| Lasiocarpine | *Heliotropium curassavicum* |
| Acetyllasiocarpine | *Heliotropium europaeum* |
| Heleurine | *Heliotropium europaeum* |
| Heliotropium europaeum Al-kaloid | *Heliotropium europaeum* |
| Lasiocarpine N-oxide | *Heliotropium europaeum* |
| Supinine | *Heliotropium europaeum* |
| Acetylindicine | *Heliotropium indicum* |
| Heleurine | *Heliotropium indicum* |
| Indicine N-oxide | *Heliotropium indicum* |
| Indicine$ | *Heliotropium indicum* |
| Lasiocarpine | *Heliotropium indicum* |
| Helifoline | *Heliotropium ovalifolium* |
| Heliscabine | *Heliotropium scabrum* |
| 1,2-Epoxy-1-hydroxymethyl-pyrrolizidine | *Heliotropium subulatum* |
| 7-Angelylheliotridine trachelanthate | *Heliotropium supinum* |
| 7-Angelylheliotridine viridiflorate | *Heliotropium supinum* |
| Supinine | *Heliotropium supinum* |
| Celliamine | *Helleborus viridis* |
| Helleborus Alkaloid V | *Helleborus viridis* |
| Fulvanine A | *Hemerocallis fulva* |
| Fulvanine B | *Hemerocallis fulva* |
| Fulvanine C | *Hemerocallis fulva* |
| Fulvanine D | *Hemerocallis fulva* |
| Fulvanine E | *Hemerocallis fulva* |
| Dehydrothalicarpine | *Hernandia ovigera* |
| N-Methylcorydaldine | *Hernandia ovigera* |
| Ovigerine | *Hernandia ovigera* |
| Oxocrebanine | *Hernandia ovigera* |
| Oxothalicarpine | *Hernandia ovigera* |
| Cannabisin H | *Hibiscus cannabinus* |
| Grossamide K | *Hibiscus cannabinus* |
| Daphniphylline | *Hibiscus sabdariffa* |
| Canthin-6-one | *Hibiscus syriacus* |
| Holafebrine | *Holarrhena mitis* |
| Holarrhimine | *Holarrhena mitis* |
| Mitiphylline | *Holarrhena mitis* |
| Kurchinine | *Holarrhena pubescens* |
| Hordatine A | *Hordeum jubatum* |
| 4-Hydroxyphenethylamine | *Hordeum vulgare* |
| Hordatine A | *Hordeum vulgare* |
| Hordatine B | *Hordeum vulgare* |
| Hordatine B | *Hordeum vulgare* |
| Hordenine | *Hordeum vulgare* |
| N-Dinorgramine | *Hordeum vulgare* |
| N-Norgramine | *Hordeum vulgare* |
| Tyramine | *Hordeum vulgare* |
| 3,5-Didecanoyl-1,4-dihydro-4-nonylpyridine | *Houttuynia cordata* |
| 3-Decanoyl-5-dodecanoyl-1,4-dihydro-4-nonylpyridine | *Houttuynia cordata* |
| 3-Nonyl-1H-pyrazole | *Houttuynia cordata* |
| 5-Decanoyl-2-nonylpyridine | *Houttuynia cordata* |
| Abereamine 1 | *Hunteria umbellata* |
| Abereamine 4 | *Hunteria umbellata* |
| Erinicine | *Hunteria umbellata* |
| O-Acetylcorymine | *Hunteria umbellata* |
| 16-Descarbomethoxy- 16,17-dihydro-17-hydroxy-19-epiajmalicine | *Hunteria zeylanica* |
| 16-Epi-10-hydroxyaffinine | *Hunteria zeylanica* |
| 3-Epidihydrocorymine 3- acetate | *Hunteria zeylanica* |
| 3-Epidihydrocorymine 17-acetate | *Hunteria zeylanica* |
| Coryzeylamine | *Hunteria zeylanica* |
| Deformylcoryzeylamine | *Hunteria zeylanica* |
| Desformocorymine | *Hunteria zeylanica* |
| Dihydrocorynantheol | *Hunteria zeylanica* |
| Fluorocarpamine | *Hunteria zeylanica* |
| Geissoschizol | *Hunteria zeylanica* |
| Hunteriatryptamine | *Hunteria zeylanica* |
| Isocorymine | *Hunteria zeylanica* |
| Isoeburnamine | *Hunteria zeylanica* |
| Ndemethylcorymine | *Hunteria zeylanica* |
| Norisocorymine | *Hunteria zeylanica* |
| Norpleiomutine | *Hunteria zeylanica* |
| O-Methyleburnamine | *Hunteria zeylanica* |
| a-Belladonnine | *Hyoscyamus niger* |
| b-Belladonnine | *Hyoscyamus niger* |
| Cannabisin G | *Hyoscyamus niger* |
| Cuscohygrine | *Hyoscyamus niger* |
| Hyoscyamide | *Hyoscyamus niger* |
| Hypecoleptopine | *Hypecoum leptocarpum* |
| Hyperectine | *Hypecoum leptocarpum* |
| Isohyperectine | *Hypecoum leptocarpum* |
| Leptocarpinine | *Hypecoum leptocarpum* |
| Leptopidine | *Hypecoum leptocarpum* |
| Leptopidinine | *Hypecoum leptocarpum* |
| Leptopine | *Hypecoum leptocarpum* |
| Leptopinine | *Hypecoum leptocarpum* |
| Procumbine | *Hypecoum leptocarpum* |
| 13-Oxoprotopine | *Hypecoum procumbens* |
| Hypecumine | *Hypecoum procumbens* |
| Procumbine | *Hypecoum procumbens* |
| Indigotin$ | *Indigofera tinctoria* |
| Cornigerine | *Iphigenia indica* |
| Merenderine | *Iphigenia indica* |
| Ipalbidine | *Ipomoea alba* |
| Ipalbine | *Ipomoea alba* |
| Ipanguline B1 | *Ipomoea hederifolia* |
| Ipanguline C1 | *Ipomoea hederifolia* |
| Ipanguline D3 | *Ipomoea hederifolia* |
| Ipanguline D9 | *Ipomoea hederifolia* |
| Ipanguline A3 | *Ipomoea hederifolia* |
| Ipanguline C2 | *Ipomoea hederifolia* |
| Ipanguline C4 | *Ipomoea hederifolia* |
| Isoipanguline B2 | *Ipomoea hederifolia* |
| Isoipanguline C2 | *Ipomoea hederifolia* |
| Isoipanguline D3 | *Ipomoea hederifolia* |
| Isoipanguline A1 | *Ipomoea hederifolia* |
| Isoipanguline B1 | *Ipomoea hederifolia* |
| Isoipanguline C1 | *Ipomoea hederifolia* |
| Isoipanguline D10 | *Ipomoea hederifolia* |
| Isoipanguline D10 | *Ipomoea quamoclit* |
| (+-)-Chanoclavine II | *Ipomoea violacea* |
| Festuclavine | *Ipomoea violacea* |
| 1H-Indole-2,5-diol | *Isatis tinctoria* |
| Isatan A | *Isatis tinctoria* |
| Isatan C | *Isatis tinctoria* |
| Isatan B | *Isatis tinctoria* |
| Sulfoglucobrassicin | *Isatis tinctoria* |
| Isopyruthaldine | *Isopyrum thalictroides* |
| Isopyruthaline | *Isopyrum thalictroides* |
| Isopythaldine | *Isopyrum thalictroides* |
| Isothalicrine | *Isopyrum thalictroides* |
| Isothalictrine | *Isopyrum thalictroides* |
| Isothalirine | *Isopyrum thalictroides* |
| Pseudocolumbamine | *Isopyrum thalictroides* |
| Columbamine | *Jateorhiza palmata* |
| Palmatine | *Jateorhiza palmata* |
| Kopsamine | *Kopsia flavida* |
| Methyl 11,12-dimethoxychanofruticosinate | *Kopsia flavida* |
| Methyl 12-methoxychanofruticosinate | *Kopsia flavida* |
| Methyl 12-methoxy-N1-demethoxycarbonylchanofruticosinate | *Kopsia flavida* |
| Prunifoline D | *Kopsia flavida* |
| Fruticosamine | *Kopsia fruticosa* |
| Fruticosine | *Kopsia fruticosa* |
| Kopsifoline B | *Kopsia fruticosa* |
| Kopsifoline C | *Kopsia fruticosa* |
| Kopsifoline D | *Kopsia fruticosa* |
| Kopsifoline E | *Kopsia fruticosa* |
| Kopsifoline A | *Kopsia fruticosa* |
| Kopsorinine | *Kopsia fruticosa* |
| Mersicarpine | *Kopsia fruticosa* |
| Lagerine | *Lagerstroemia indica* |
| 5-Oxo-2(5H)-isoxazoleacetic acid | *Lathyrus odoratus* |
| 5-Oxo-2(5H)-isoxazolepropanenitrile | *Lathyrus odoratus* |
| Homoagmatine | *Lathyrus sativus* |
| Cassythicine | *Laurus nobilis* |
| Cryptodorine | *Laurus nobilis* |
| Isodomesticine | *Laurus nobilis* |
| Launobine | *Laurus nobilis* |
| Nandigerine | *Laurus nobilis* |
| 6-Bromohypaphorine | *Lens culinaris* |
| O-Acetylethanolamine | *Lens culinaris* |
| (4-Hydroxybutyl)guanidine,9CI | *Leonurus sibiricus* |
| Leonuridine | *Leonurus sibiricus* |
| Leonurine | *Leonurus sibiricus* |
| Lepidine B | *Lepidium sativum* |
| Lepidine C | *Lepidium sativum* |
| Lepidine D | *Lepidium sativum* |
| Mimoside | *Leucaena leucocephala* |
| 21-O-Methylleuconolam | *Leuconotis eugenifolia* |
| Epileuconolam | *Leuconotis eugenifolia* |
| Leuconolam | *Leuconotis eugenifolia* |
| 1,5-Dihydro-5-hydroxy-3-methyl-1-(5-oxo-2-pyrrolidinyl)-2Hpyrrol- 2-one | *Lilium candidum* |
| Jatropham | *Lilium martagon* |
| Acidissiminin | *Limonia acidissima* |
| Acidissimininepoxide | *Limonia acidissima* |
| Acidissiminol | *Limonia acidissima* |
| Acidissiminol epoxide | *Limonia acidissima* |
| Nb-Acetyl-Nb-methyltryptamine | *Limonia acidissima* |
| Linamarin | *Linum usitatissimum* |
| Linusitamarin | *Linum usitatissimum* |
| Nervosine | *Liparis nervosa* |
| (+)-Isolaureline | *Liriodendron tulipifera* |
| 10-Hydroxy-1,2,9-trimethoxy- aporphine | *Liriodendron tulipifera* |
| Corunnine | *Liriodendron tulipifera* |
| Dehydroglaucine | *Liriodendron tulipifera* |
| Dehydroisolaureline | *Liriodendron tulipifera* |
| Dehydroroemerine | *Liriodendron tulipifera* |
| Liriodendronine | *Liriodendron tulipifera* |
| Oxoglaucine | *Liriodendron tulipifera* |
| Oxopurpureine | *Liriodendron tulipifera* |
| Predicentrine | *Liriodendron tulipifera* |
| Lithospermoside | *Lithospermum officinale* |
| 8-O-Methyloblongine | *Litsea cubeba* |
| Litcubine | *Litsea cubeba* |
| Litebamine | *Litsea cubeba* |
| Oblongine$ | *Litsea cubeba* |
| Cassythicine | *Litsea glutinosa* |
| Litseferine | *Litsea glutinosa* |
| (-)-Lelobanidine II | *Lobelia nicotianaefolia* |
| (-)-Lelobanidine III | *Lobelia nicotianaefolia* |
| 13-Acetoxylupanine | *Lupinus albus* |
| 13a-Hydroxymultiflorine | *Lupinus albus* |
| 13-Benzoyloxylupanine | *Lupinus albus* |
| 13-Butyryloxylupanine | *Lupinus albus* |
| 13-Isobutyryloxylupanine | *Lupinus albus* |
| 13-Isovaleroyloxylupanine | *Lupinus albus* |
| 13-Propanoyloxylupanine | *Lupinus albus* |
| 13-Valeroyloxylupanine | *Lupinus albus* |
| 15-Hydroxy-17-oxolupanine | *Lupinus albus* |
| Albine | *Lupinus albus* |
| Alkaloid LC2 | *Lupinus albus* |
| Angustifoline | *Lupinus albus* |
| Dehydroangustifoline | *Lupinus albus* |
| Lupanine N-oxide | *Lupinus albus* |
| Multiflorine | *Lupinus albus* |
| Nuttalline acetate | *Lupinus albus* |
| 13-Benzoyloxylupanine | *Lupinus angustifolius* |
| 13-Butyryloxylupanine | *Lupinus angustifolius* |
| 13-Caproyloxylupanine | *Lupinus angustifolius* |
| 13-Epimethoxylupanine | *Lupinus angustifolius* |
| 13-Propanoyloxylupanine | *Lupinus angustifolius* |
| 17-Oxolupanine | *Lupinus angustifolius* |
| Isoangustifoline | *Lupinus angustifolius* |
| Nuttalline acetate | *Lupinus angustifolius* |
| Alkaloid LV3 | *Lupinus digitatus* |
| Alkaloid LV4 | *Lupinus digitatus* |
| Epilupinine | *Lupinus digitatus* |
| (4'-a-L-Rhamnosyloxycinnamoyl)lupinine | *Lupinus luteus* |
| (4'-b-D-Glucopyranosyloxycinnamoyl)lupinine | *Lupinus luteus* |
| (4-Hydroxycinnamoyl)lupinine | *Lupinus luteus* |
| (4'-O-b-D-Glucopyranosyloxy-3'-methoxycinnamoyl)lupinine | *Lupinus luteus* |
| v-Feruloyloxylupinane | *Lupinus luteus* |
| Lyciumin B | *Lycium barbarum* |
| Lyciumin D | *Lycium barbarum* |
| 4-Coumaroylputrescine | *Lycopersicon esculentum* |
| Esculeoside B | *Lycopersicon esculentum* |
| Esculeoside C | *Lycopersicon esculentum* |
| 3-(3,4-Dihydroxyphenyl)pro-panoic acid | *Lycopodium clavatum* |
| Acetyldihydrolycopodine | *Lycopodium clavatum* |
| Acetylfawcettiine | *Lycopodium clavatum* |
| Acetylfawcettiine | *Lycopodium clavatum* |
| Acetyllycoclavine | *Lycopodium clavatum* |
| Flabelliformine | *Lycopodium clavatum* |
| Lucidioline | *Lycopodium clavatum* |
| Lycoclavine | *Lycopodium clavatum* |
| Lycodine | *Lycopodium clavatum* |
| Lycopodium clavatum Alkaloid | *Lycopodium clavatum* |
| Lycovatine A | *Lycopodium clavatum* |
| 9-Norpluviine | *Lycoris radiata* |
| Lycorenine | *Lycoris radiata* |
| Pluviine | *Lycoris radiata* |
| Pseudolycorine | *Lycoris radiata* |
| Ntrans- Caffeoyltyramine | *Mangifera indica* |
| Betonicine | *Marrubium vulgare* |
| Emarginatine A | *Maytenus emarginata* |
| Emarginatinine | *Maytenus emarginata* |
| Wilforine | *Maytenus senegalensis* |
| Homostachydrine | *Medicago sativa* |
| N6-Methylagmatine | *Medicago sativa* |
| 4-Hydroxyphenylacetic acid | *Melilotus officinalis* |
| Luteolin | *Melissa officinalis* |
| Adouetine Y' | *Melochia corchorifolia* |
| Franganine | *Melochia corchorifolia* |
| Frangufoline | *Melochia corchorifolia* |
| Melochicorine | *Melochia corchorifolia* |
| Leucenine | *Mimosa pudica* |
| Mimopudine | *Mimosa pudica* |
| Mimoside | *Mimosa pudica* |
| Corynantheidol | *Mitragyna parvifolia* |
| Corynantheine | *Mitragyna parvifolia* |
| Dihydrocorynantheol N-oxide | *Mitragyna parvifolia* |
| Hirsuteine | *Mitragyna parvifolia* |
| Hirsutine | *Mitragyna parvifolia* |
| Speciophylline N-oxide | *Mitragyna parvifolia* |
| Uncarine E | *Mitragyna parvifolia* |
| Uncarine F N-oxide | *Mitragyna parvifolia* |
| Charine | *Momordica charantia* |
| Benzylamine | *Moringa oleifera* |
| Niazicin A | *Moringa oleifera* |
| Niazimicin A | *Moringa oleifera* |
| Niazimicin B | *Moringa oleifera* |
| Niazimin A | *Moringa oleifera* |
| Niazimin B | *Moringa oleifera* |
| Niaziminin A | *Moringa oleifera* |
| Niazinin B | *Moringa oleifera* |
| Niazirin | *Moringa oleifera* |
| Niazirinin | *Moringa oleifera* |
| Lycopene | *Moringa peregrina* |
| 2-O-a-D-Galactopyranosyl- 1-deoxynojirimycin | *Morus alba* |
| 2-O-a-D-Glucopyranosyl- 1-deoxynojirimycin | *Morus alba* |
| 2-O-b-D-Glucopyranosyl- 1-deoxynojirimycin | *Morus alba* |
| 3-O-a-D-Glucopyranosyl- 1-deoxynojirimycin | *Morus alba* |
| 3-O-b-D-Glucopyranosyl- 1-deoxynojirimycin | *Morus alba* |
| 4-Epifagomine | *Morus alba* |
| 4-O-a-D-Glu-copyranosyl-1-deoxynojirimycin | *Morus alba* |
| 4-O-b-D-Glucopyranosyl- 1-deoxynojirimycin | *Morus alba* |
| 5-Hydroxy-2-piperidinecar- boxylic acid | *Morus alba* |
| 6-O-b-D-Glucopyranosyl- 1-deoxynojirimycin | *Morus alba* |
| Morusimic acid A | *Morus alba* |
| Morusimic acid C | *Morus alba* |
| Morusimic acid D | *Morus alba* |
| Morusimic acid E | *Morus alba* |
| Morusimic acid F | *Morus alba* |
| N-Methyl-1-deoxynojirimycin | *Morus alba* |
| Mucuadinine | *Mucuna pruriens* |
| Mucuadininine | *Mucuna pruriens* |
| Mucuna pruriens Alkaloid P | *Mucuna pruriens* |
| Mucuna pruriens Alkaloid Q | *Mucuna pruriens* |
| Mucuna pruriens Alkaloid S | *Mucuna pruriens* |
| Mucunadine | *Mucuna pruriens* |
| Mucunine | *Mucuna pruriens* |
| Prurieninine | *Mucuna pruriens* |
| (+)-Mahanimbine | *Murraya koenigii* |
| (+-)-Mahanimbine | *Murraya koenigii* |
| Bicyclomahanimbicine | *Murraya koenigii* |
| Bicyclomahanimbine | *Murraya koenigii* |
| Bikoeniquinone A | *Murraya koenigii* |
| Bismurrayaquinone A | *Murraya koenigii* |
| Mahanimbilol | *Murraya koenigii* |
| Mukoenine B | *Murraya koenigii* |
| Murrastifoline F | *Murraya koenigii* |
| Murrayacine | *Murraya koenigii* |
| Murrayanine | *Murraya koenigii* |
| Murrayanol$ | *Murraya koenigii* |
| De-N-methylacronycine | *Murraya paniculata* |
| Isomurralonginol nicotinate | *Murraya paniculata* |
| Murrayacarine | *Murraya paniculata* |
| Noracronycine | *Murraya paniculata* |
| Dopamine | *Musa sapientum* |
| 4-Methoxybenzoic acid | *Naravelia zeylanica* |
| Maritidine | *Narcissus tazetta* |
| Narcisine | *Narcissus tazetta* |
| O-Methylmaritidine | *Narcissus tazetta* |
| Precriwelline | *Narcissus tazetta* |
| 10-Hydroxyangustine | *Nauclea orientalis* |
| 3,14-Dihydroangustoline | *Nauclea orientalis* |
| Dehydroanonaine | *Nelumbo nucifera* |
| Dehydronuciferine | *Nelumbo nucifera* |
| Dehydroroemerine | *Nelumbo nucifera* |
| Neferine | *Nelumbo nucifera* |
| Pronuciferine | *Nelumbo nucifera* |
| Nepetalactam | *Nepeta cataria* |
| 2,3?-Bipyridine | *Nicotiana tabacum* |
| 2,3'-Bipyrrolidine | *Nicotiana tabacum* |
| 3-Phenylpropanoic acid | *Nicotiana tabacum* |
| 4-Coumaroylputrescine | *Nicotiana tabacum* |
| Cotinine | *Nicotiana tabacum* |
| N1,N4-Dicaffeoylputrescine | *Nicotiana tabacum* |
| N1,N4-Diferuloylputrescine | *Nicotiana tabacum* |
| N1,N4-Di-p-coumaroylputrescine | *Nicotiana tabacum* |
| Nicotine | *Nicotiana tabacum* |
| Nicotine 1'-N-oxide | *Nicotiana tabacum* |
| Paucine | *Nicotiana tabacum* |
| Damascenine | *Nigella damascena* |
| Damascinine | *Nigella damascena* |
| Isosalsolidine | *Nigella sativa* |
| Nigellidine | *Nigella sativa* |
| Nigellimine N-oxide | *Nigella sativa* |
| Nympheine | *Nymphaea alba* |
| Jasminine$ | *Olea paniculata* |
| 10-Methoxycamptothecin | *Ophiorrhiza mungos* |
| 9-Methoxycamptothecin | *Ophiorrhiza mungos* |
| 1H-Indol-3-ylacetyl-myo-in- ositol | *Oryza sativa* |
| N-Jasmonoylleucine | *Oryza sativa* |
| Pachygonine | *Pachygone ovata* |
| Trilobine | *Pachygone ovata* |
| Pidolic acid | *Panax ginseng* |
| Alkaloid PAR 2 | *Papaver argemone* |
| Alkaloid PAR 3 | *Papaver argemone* |
| Mecambrine | *Papaver dubium* |
| Oxyhydrastinine | *Papaver dubium* |
| 13-Oxomuramine | *Papaver nudicaule* |
| 5-Hydroxy-2-(hydroxy-methyl)pyridine | *Papaver nudicaule* |
| 8,14-Dihydroamurine | *Papaver nudicaule* |
| Alborine | *Papaver nudicaule* |
| Alkaloid PO4 | *Papaver nudicaule* |
| Amurensine | *Papaver nudicaule* |
| Amuroline | *Papaver nudicaule* |
| Amuronine | *Papaver nudicaule* |
| Muramine | *Papaver nudicaule* |
| Nudaurine | *Papaver nudicaule* |
| Nudaurine | *Papaver nudicaule* |
| Nudaurine | *Papaver nudicaule* |
| 8,9-Dihydroisoorientalinone | *Papaver orientale* |
| Alkaloid Or2 | *Papaver orientale* |
| Alpinigenine | *Papaver orientale* |
| Bracteoline | *Papaver orientale* |
| Orientalidine | *Papaver orientale* |
| Alkaloid PP1 | *Papaver pavoninum* |
| Adlumidiceine | *Papaver rhoeas* |
| Dehydroroemerine | *Papaver rhoeas* |
| Papaver Alkaloid | *Papaver rhoeas* |
| Papaver rhoeas Alkaloid | *Papaver rhoeas* |
| 13-Oxocryptopine | *Papaver somniferum* |
| 2-Hydroxy-4-quinolinecar- boxylic acid | *Papaver somniferum* |
| Bismorphine A | *Papaver somniferum* |
| Bismorphine B | *Papaver somniferum* |
| Codeine | *Papaver somniferum* |
| Codeine N-oxide | *Papaver somniferum* |
| Hydrocotarnine | *Papaver somniferum* |
| Laudanidine | *Papaver somniferum* |
| Laudanine | *Papaver somniferum* |
| Morphine N-oxide | *Papaver somniferum* |
| Narceine | *Papaver somniferum* |
| Narceinone | *Papaver somniferum* |
| Neopine | *Papaver somniferum* |
| Noscapine | *Papaver somniferum* |
| 17-Methylparsonsianidine | *Parsonsia laevigata* |
| Parsonsianine | *Parsonsia laevigata* |
| Harmine | *Passiflora edulis* |
| 6-O-(1,3-Dihydroxyisopropyl) inosine | *Pedicularis longiflora* |
| 2-(3-Hydroxypropyl)-4(3H)- quinazolinone | *Peganum harmala* |
| Alkaloid YC2 | *Peganum harmala* |
| Deoxypeganidine | *Peganum harmala* |
| Dihydroruine | *Peganum harmala* |
| Dipegine | *Peganum harmala* |
| Dipeginol | *Peganum harmala* |
| Harmalol | *Peganum harmala* |
| Harmine | *Peganum harmala* |
| Isopeganidine | *Peganum harmala* |
| Pegamine | *Peganum harmala* |
| Ruine | *Peganum harmala* |
| Prunasin | *Perilla frutescens* |
| Peristrophine | *Peristrophe roxburghiana* |
| Gramine | *Phalaris arundinacea* |
| 5-Hydroxy-2-piperidinecar- boxylic acid | *Phoenix dactylifera* |
| 2',3',4',6'- Tetra-O-galloylprunasin | *Phyllagathis rotundifolia* |
| 2',3',6'-Tri-Ogalloylprunasin | *Phyllagathis rotundifolia* |
| 2',6'-Di-O-galloylprunasin | *Phyllagathis rotundifolia* |
| 3',4',6'-Tri-Ogalloylprunasin | *Phyllagathis rotundifolia* |
| 3',6'-Di-O-galloylprunasin | *Phyllagathis rotundifolia* |
| 4',6'-Di-O-galloylprunasin | *Phyllagathis rotundifolia* |
| Epibubbialine | *Phyllanthus amarus* |
| Isobubbialine | *Phyllanthus amarus* |
| 2,4-Octadienoic acid | *Phyllanthus fraternus* |
| Calystegine B3 | *Physalis alkekengi* |
| Physoperuvine | *Physalis peruviana* |
| Calabacine | *Physostigma venenosum* |
| Calabatine | *Physostigma venenosum* |
| Eseramine | *Physostigma venenosum* |
| Isophysostigmine | *Physostigma venenosum* |
| N8-Norphysostigmine | *Physostigma venenosum* |
| Physostigmine | *Physostigma venenosum* |
| Physovenine | *Physostigma venenosum* |
| 2-(2-Hydroxypropyl)-6- methylpiperidine | *Picea abies* |
| Pinidinol | *Picea abies* |
| 4-Methoxy-1-vinyl-b-carboline | *Picrasma javanica* |
| 6-Hydroxydehydrocrenatine | *Picrasma javanica* |
| Dehydrocrenatine | *Picrasma javanica* |
| Picrasidine I | *Picrasma javanica* |
| Picrasidine J | *Picrasma javanica* |
| 3-Ethoxycarbonyl-b-carboline | *Picrasma quassioides* |
| 4-Hydroxy-b-carboline-1-carboxaldehyde | *Picrasma quassioides* |
| 4-Hydroxy-b-carboline-1-carboxylic acid | *Picrasma quassioides* |
| b-Carboline-1,3,4-trione | *Picrasma quassioides* |
| b-Carboline-1-propanoic acid | *Picrasma quassioides* |
| Dehydrocrenatidine | *Picrasma quassioides* |
| Kumujansine | *Picrasma quassioides* |
| Kumujansine B | *Picrasma quassioides* |
| Kumujian C | *Picrasma quassioides* |
| Picrasidine A | *Picrasma quassioides* |
| Picrasidine F | *Picrasma quassioides* |
| Picrasidine I | *Picrasma quassioides* |
| Picrasidine J | *Picrasma quassioides* |
| Picrasidine L | *Picrasma quassioides* |
| Picrasidine O | *Picrasma quassioides* |
| Picrasidine P | *Picrasma quassioides* |
| 4-Methoxybenzoic acid | *Pimpinella anisum* |
| 1-Cinnamoyl-1H-pyrrole | *Piper argyrophyllum* |
| 4,5-Dihydropiperlonguminine | *Piper guineense* |
| Cycloguineense A | *Piper guineense* |
| Cycloguineense B | *Piper guineense* |
| N-Isobutyl- 2,4-eicosadienamide | *Piper guineense* |
| N-Isobutyl-2,4-octadecadienamide | *Piper guineense* |
| 3,4-Methylenedioxybenzoic acid | *Piper longum* |
| 4,5-Dihydropiperlonguminine | *Piper longum* |
| Aristolodione | *Piper longum* |
| Piperlongine | *Piper longum* |
| Pipernonaline | *Piper longum* |
| Piplartine | *Piper longum* |
| Tetrahydropiperine | *Piper longum* |
| 1-Piperidinecarboxaldehyde | *Piper nigrum* |
| 2,4-Dodecadienoic acid isobutylamide | *Piper nigrum* |
| Brachyamide B | *Piper nigrum* |
| Chavicine | *Piper nigrum* |
| Coumaperine | *Piper nigrum* |
| Dipiperamide A | *Piper nigrum* |
| Feruperine | *Piper nigrum* |
| Isochavicine | *Piper nigrum* |
| Isopiperolein B | *Piper nigrum* |
| Nigramide C | *Piper nigrum* |
| Nigramide D | *Piper nigrum* |
| Nigramide E | *Piper nigrum* |
| Nigramide G | *Piper nigrum* |
| Nigramide J | *Piper nigrum* |
| Nigramide M | *Piper nigrum* |
| Nigramide N | *Piper nigrum* |
| N-trans-Feruloyltyramine | *Piper nigrum* |
| Piperamide-C5:1 (2E) | *Piper nigrum* |
| Piperamide-C7:1 (6E) | *Piper nigrum* |
| Piperamide-C9:3 (2E,4E,8E) | *Piper nigrum* |
| Pipercitine | *Piper nigrum* |
| Pipercyclamide | *Piper nigrum* |
| Pipercyclobutanamide B | *Piper nigrum* |
| Piperettine | *Piper nigrum* |
| Pipericine | *Piper nigrum* |
| Piperidine | *Piper nigrum* |
| Piperine | *Piper nigrum* |
| Piperolein A | *Piper nigrum* |
| Piperolein B | *Piper nigrum* |
| Pipertipine | *Piper nigrum* |
| Pipsaeedine | *Piper nigrum* |
| Tricholein | *Piper nigrum* |
| 2-Methoxy-4,5-methylenedioxycinnamoyl isobutylamide | *Piper peepuloides* |
| Peepuloidine | *Piper peepuloides* |
| 1-(5-Methyl-3-pyridinyl)-1-decanone | *Piper retrofractum* |
| 2,4,12-Octadecatrienoic acid | *Piper retrofractum* |
| 2,4,14-Eicosatrienoic acid isobutylamide | *Piper retrofractum* |
| N-(2,14-Eicosadienoyl)piperidine | *Piper retrofractum* |
| N-(2,4-Eicosadienoyl)-piperidine | *Piper retrofractum* |
| Pipereicosalidine | *Piper retrofractum* |
| Pipnoohine | *Piper retrofractum* |
| 2,4-Decadienoic acid | *Piper sarmentosum* |
| N-(3-Phenylpropanoyl)pyrrole | *Piper sarmentosum* |
| Sarmentine | *Piper sarmentosum* |
| Sarmentosine | *Piper sarmentosum* |
| 2,4-Decadienoic acid isobu-tylamide | *Piper sylvaticum* |
| Piperlonguminine | *Piper sylvaticum* |
| Piplartine | *Piper sylvaticum* |
| Cyclopiperstachine | *Piper trichostachyon* |
| Cyclostachine A | *Piper trichostachyon* |
| Piperstachine | *Piper trichostachyon* |
| 1H-Indole-3-carboxylic acid | *Pisum sativum* |
| 2-Methoxy-3-(1-methylpropyl)pyrazine | *Pisum sativum* |
| 4-Chloro-1H-indole-3-acetic acid | *Pisum sativum* |
| 4-Hydroxyphenethylamine | *Pisum sativum* |
| 5-Oxo-2(5H)-isoxazolepropanenitrile | *Pisum sativum* |
| Dihydromaleimide b-D-glucoside | *Pisum sativum* |
| Pisatoside | *Pisum sativum* |
| Tyramine | *Pisum sativum* |
| Indicamine$ | *Plantago Ovata* |
| N-Benzoyltyramine methyl ether | *Pleiospermium alatum* |
| N-Homoveratroylhomovera-trylamine | *Pleiospermium alatum* |
| Oppositine A | *Pleurostylia opposita* |
| Isooncodine | *Polyalthia longifolia* |
| O-Methylbulbocapnine a-N-oxide | *Polyalthia longifolia* |
| Polyfothine | *Polyalthia longifolia* |
| Indicaxanthin | *Portulaca grandiflora* |
| Mesembryanthemins | *Portulaca grandiflora* |
| Neobetanin | *Portulaca grandiflora* |
| Portulacaxanthin III | *Portulaca grandiflora* |
| Oleracein A | *Portulaca oleracea* |
| Oleracein D | *Portulaca oleracea* |
| Oleracein C | *Portulaca oleracea* |
| Oleracins | *Portulaca oleracea* |
| 1-(N-Methyl-2-piperidinyl)- 2-butanone | *Pratia nummularia* |
| 3''-Oxojuliflorine | *Prosopis juliflora* |
| 3-Oxojuliprosine | *Prosopis juliflora* |
| 3'-Oxojuloprosine | *Prosopis juliflora* |
| Isojuliprosine | *Prosopis juliflora* |
| Julifloridine | *Prosopis juliflora* |
| Juliflorine | *Prosopis juliflora* |
| Juliprosine | *Prosopis juliflora* |
| N-Methyljulifloridine | *Prosopis juliflora* |
| 6'-O-Galoylsambunigrin | *Prunus persica* |
| Persicaside | *Prunus persica* |
| Psychotrine | *Psychotria ipecacuanha* |
| Pelletierine | *Punica granatum* |
| N1,N4-Dicaffeoylputrescine | *Pyrus communis* |
| N1,N4-Di-p-coumaroylputrescine | *Pyrus communis* |
| 1-Methoxy-1H-indole-3-carboxaldehyde, 9CI | *Raphanus sativus* |
| Biotin | *Raphanus sativus* |
| Caulilexin C | *Raphanus sativus* |
| Olomoucine | *Raphanus sativus* |
| Raphanusamide | *Raphanus sativus* |
| 2-Hydroxybenzylamine | *Reseda odorata* |
| Decursivine | *Rhaphidophora decursiva* |
| 1,2-Dehydroaspidospermidine N-oxide | *Rhazya stricta* |
| 16-Epi-Z-isositsirikine | *Rhazya stricta* |
| Akuammidine | *Rhazya stricta* |
| Alkaloid D | *Rhazya stricta* |
| Antirhine | *Rhazya stricta* |
| Aspidospermidose | *Rhazya stricta* |
| Aspidospermiose | *Rhazya stricta* |
| Bharhingine | *Rhazya stricta* |
| Bhimberine | *Rhazya stricta* |
| Decarbomethoxytetrahydro-secodine | *Rhazya stricta* |
| Dihydrocorynantheol | *Rhazya stricta* |
| Dihydroeburnamenine | *Rhazya stricta* |
| Geissoschizine | *Rhazya stricta* |
| N-Methylleuconolam | *Rhazya stricta* |
| O-Acetylisositsirikine | *Rhazya stricta* |
| Picralinal | *Rhazya stricta* |
| Sewarine | *Rhazya stricta* |
| Rhizophorine | *Rhizophora mucronata* |
| 5-O-Feruloylnigrumin | *Ribes nigrum* |
| 5-O-p-Coumaroylnigrumin | *Ribes nigrum* |
| Flazine methyl ether | *Ribes nigrum* |
| Sarmentosin$ | *Ribes rubrum* |
| N-Demethylricinine | *Ricinus communis* |
| Rorifone | *Rorippa montana* |
| Rosmarinus officinalis Alkaloid 2 | *Rosmarinus officinalis* |
| 3-Hydroxy-1,4-naphthoqui- none-2-carboxylic acid | *Rubia cordifolia* |
| 5-Methoxydictamnine | *Ruta chalepensis* |
| 8-Methoxytaifine | *Ruta chalepensis* |
| Chaloridone | *Ruta chalepensis* |
| Taifine | *Ruta chalepensis* |
| 1-Hydroxy-10-methylacridone | *Ruta graveolens* |
| 2-Dodecyl-4-hydroxyquino-line | *Ruta graveolens* |
| 2-Undecyl-4(1H)-quinolinone N-oxide | *Ruta graveolens* |
| 4-Hydroxy-2-undecylquino- line | *Ruta graveolens* |
| Arborine | *Ruta graveolens* |
| Furofoline | *Ruta graveolens* |
| Graveolinine | *Ruta graveolens* |
| Kokusaginine | *Ruta graveolens* |
| Platydesminium | *Ruta graveolens* |
| Pretazettine | *Ruta graveolens* |
| Pteleine | *Ruta graveolens* |
| 2-Acetyl-3-methylindole | *Salvadora persica* |
| Benzylurea | *Salvadora persica* |
| Sambucus nigra Degraded cyanogenic glycosides | *Sambucus nigra* |
| 1-(2-Furanylmethyl)-1H-pyrrole | *Santalum album* |
| AndrographidoidsA | *Santolina insularis* |
| Cirsimaritin | *Santolina insularis* |
| Papraline | *Santolina insularis* |
| Parthenolide | *Santolina insularis* |
| Pyridoxine | *Santolina insularis* |
| Tanetin | *Santolina insularis* |
| Apparicine | *Sapium sebiferum* |
| 2-Hydroxysalignarine E | *Sarcococca saligna* |
| 3-Epipachysamine H | *Sarcococca saligna* |
| 5,6-Dihydrosarconidine | *Sarcococca saligna* |
| Epipachysamine D | *Sarcococca saligna* |
| E-Salignone | *Sarcococca saligna* |
| Isosaracodine | *Sarcococca saligna* |
| N3-Demethylsaracodine | *Sarcococca saligna* |
| Saligcinnamide | *Sarcococca saligna* |
| Salignamine | *Sarcococca saligna* |
| Salignarine A | *Sarcococca saligna* |
| Salignarine B | *Sarcococca saligna* |
| Salignarine D | *Sarcococca saligna* |
| Salignarine E | *Sarcococca saligna* |
| Salignarine F | *Sarcococca saligna* |
| Salignenamide A | *Sarcococca saligna* |
| Salignenamide B | *Sarcococca saligna* |
| Salignenamide F | *Sarcococca saligna* |
| Salignenamide C | *Sarcococca saligna* |
| Salonine A | *Sarcococca saligna* |
| salonine B | *Sarcococca saligna* |
| Saracocine | *Sarcococca saligna* |
| Saracodine | *Sarcococca saligna* |
| Saracorine | *Sarcococca saligna* |
| Sarcosaligmine | *Sarcococca saligna* |
| 1H-Indole-2,3-dione, 9CI | *Schizophyllum commune* |
| Isatin | *Schizophyllum commune* |
| 15b-Ethoxy- 14,15-dihydroviroallosecurinine | *Securinega suffruticosa* |
| Allosecurinine | *Securinega suffruticosa* |
| Phyllantidine | *Securinega suffruticosa* |
| Secuamamine D | *Securinega suffruticosa* |
| Dihydronorsecurinine | *Securinega virosa* |
| c-Ephedrine | *Sida cordifolia* |
| Ephedrine | *Sida cordifolia* |
| AmyrisinA | *Silybum Marianum* |
| AmyrisinB | *Silybum Marianum* |
| AmyrisinC | *Silybum Marianum* |
| Anthraxin | *Silybum Marianum* |
| Cannflavin | *Silybum Marianum* |
| Derrisin | *Silybum Marianum* |
| Dihydroxy-348-trimethoxyxanthone | *Silybum Marianum* |
| Diprenyleriodictyol | *Silybum Marianum* |
| DoitunggarcinoneC | *Silybum Marianum* |
| Hydroxymunduserone | *Silybum Marianum* |
| Isomangostin | *Silybum Marianum* |
| Isopomiferin | *Silybum Marianum* |
| IsosilybinA | *Silybum Marianum* |
| IsosilybinB | *Silybum Marianum* |
| Mearnsetin | *Silybum Marianum* |
| Mundulinol | *Silybum Marianum* |
| OphiopogonanoneG | *Silybum Marianum* |
| SchizolaenoneB | *Silybum Marianum* |
| SilybinA | *Silybum Marianum* |
| SilybinB | *Silybum Marianum* |
| SilybinC | *Silybum Marianum* |
| SilybinD | *Silybum Marianum* |
| Silydianin | *Silybum Marianum* |
| silydianinB | *Silybum Marianum* |
| TanetinB | *Silybum Marianum* |
| Taxifolin | *Silybum Marianum* |
| TomentodiplaconeB | *Silybum Marianum* |
| (4-Hydroxybenzoyl) choline | *Sinapis alba* |
| 4-Hydroxybenzylamine | *Sinapis alba* |
| Sinalbin A | *Sinapis alba* |
| Sinalexin | *Sinapis alba* |
| 2'-O-Acetyledulinine | *Skimmia laureola* |
| O-Acetylptelefoliarine | *Skimmia laureola* |
| O-Methylisoplatydesmine | *Skimmia laureola* |
| Orixiarine | *Skimmia laureola* |
| Ptelefoliarine | *Skimmia laureola* |
| Isosolanogantamine | *Solanum giganteum* |
| Solanogantamine | *Solanum giganteum* |
| Solanogantine | *Solanum giganteum* |
| 5, 7, 3', 4'-tetrahydroxyflavone | *Solanum nigrum* |
| 5, 7, 4'-trihydroxyflavone | *Solanum nigrum* |
| ApigeninB | *Solanum nigrum* |
| Caffeic Acid | *Solanum nigrum* |
| Fumaric Acid | *Solanum nigrum* |
| Potassium Sorbate | *Solanum nigrum* |
| Rhamnetin | *Solanum nigrum* |
| Solanum Alkaloid IV | *Solanum nigrum* |
| Tanaparthe | *Solanum nigrum* |
| Tanaparthe | *Solanum nigrum* |
| Solanopubamine | *Solanum pubescens* |
| 3b-Allosolanidan-3-ol | *Solanum tuberosum* |
| Metiprenaline | *Solanum tuberosum* |
| Paucine | *Solanum tuberosum* |
| Argentine | *Sophora griffithii* |
| Sophora griffithii Alkaloid B | *Sophora griffithii* |
| Epilamprolobine Noxide | *Sophora tomentosa* |
| N-Acetylcytisine | *Sophora tomentosa* |
| Dhurrin 6'-glucoside | *Sorghum bicolor* |
| Spilanthic acid | *Spilanthes acmella* |
| N-(2-Methylpropyl)-2-nonene-6,8-diynamide | *Spilanthes oleracea* |
| Spilanthic acid | *Spilanthes oleracea* |
| Turicine | *Stachys sylvatica* |
| 3-Furanmethanol | *Stellaria aquatica* |
| Isotuberostemonine | *Stemona tuberosa* |
| Corydalmine | *Stephania glabra* |
| Dehydrocorydalmine | *Stephania glabra* |
| Palmatrubine | *Stephania glabra* |
| Stephania glabra Alkaloid A | *Stephania glabra* |
| Stephania glabra Alkaloid C | *Stephania glabra* |
| Stepharine | *Stephania glabra* |
| 16-Oxoprometaphanine | *Stephania japonica* |
| Epistephanine | *Stephania japonica* |
| Hypoepistephanine | *Stephania japonica* |
| Obamegine | *Stephania japonica* |
| Oxostephanine | *Stephania japonica* |
| Oxostephasunoline | *Stephania japonica* |
| Prometaphanine | *Stephania japonica* |
| Protostephanine | *Stephania japonica* |
| Stephabyssine | *Stephania japonica* |
| Stephania japonica Alkaloid A | *Stephania japonica* |
| Stephasunoline | *Stephania japonica* |
| Steponine | *Stephania japonica* |
| Thalrugosine | *Stephania japonica* |
| Cycleanine | *Stephania rotunda* |
| a-Dihydrolysergol | *Stictocardia campanulata* |
| Elymoclavine | *Stictocardia campanulata* |
| 12-Hydroxy-11- methoxy-N-methyl-sec-pseudostrychnine | *Strychnos cinnamomifolia* |
| Pseudobrucine | *Strychnos gaultheriana* |
| Pseudostrychnine | *Strychnos gaultheriana* |
| 3-Methoxystrychnine | *Strychnos ignatii* |
| Diaboline | *Strychnos nux-vomica* |
| Geissoschizal | *Strychnos nux-vomica* |
| Isobrucine | *Strychnos nux-vomica* |
| Isostrychnine | *Strychnos nux-vomica* |
| Novacine | *Strychnos nux-vomica* |
| O-Demethylisobrucine | *Strychnos nux-vomica* |
| Protostrychnine | *Strychnos nux-vomica* |
| Pseudobrucine | *Strychnos nux-vomica* |
| Vomicine | *Strychnos nux-vomica* |
| (20S)-Dihydroantirhine | *Strychnos potatorum* |
| 1-Carbamoyl-b-carboline | *Strychnos potatorum* |
| 20-Epiantirhine | *Strychnos potatorum* |
| Akuammidine | *Strychnos potatorum* |
| b-Carboline | *Strychnos potatorum* |
| Diaboline | *Strychnos potatorum* |
| Diaboline N-oxide | *Strychnos potatorum* |
| Henningsamine | *Strychnos potatorum* |
| Nigritanine | *Strychnos potatorum* |
| Ochrolifuanine A | *Strychnos potatorum* |
| Ochrolifuanine E | *Strychnos potatorum* |
| Polyneuridine | *Strychnos potatorum* |
| 15-Hydroxyicajine | *Strychnos wallichiana* |
| Icajine | *Strychnos wallichiana* |
| N-Methyl-sec-pseudo-b-colubrine | *Strychnos wallichiana* |
| Pseudobrucine | *Strychnos wallichiana* |
| Pseudostrychnine | *Strychnos wallichiana* |
| 3-(2-Oxopropyl)coronaridine | *Tabernaemontana dichotoma* |
| 3'-Hydroxy- N4-demethylervahanine B | *Tabernaemontana dichotoma* |
| 3'-Hydroxy-N4-demethylervahanine A | *Tabernaemontana dichotoma* |
| Isomethuenine | *Tabernaemontana dichotoma* |
| Monogagaine | *Tabernaemontana dichotoma* |
| (+-)-19-Hydroxycoronaridine | *Tabernaemontana divaricata* |
| 11-Methoxy-N-methyldihydropericyclivine | *Tabernaemontana divaricata* |
| 19-Hydroxyconopharyngine | *Tabernaemontana divaricata* |
| 3S-Cyanocoronaridine | *Tabernaemontana divaricata* |
| 5-Oxocoronaridine | *Tabernaemontana divaricata* |
| 6-Oxocoronaridine | *Tabernaemontana divaricata* |
| Conolobine A | *Tabernaemontana divaricata* |
| Conolobine B | *Tabernaemontana divaricata* |
| Conophylline | *Tabernaemontana divaricata* |
| Conophyllinine | *Tabernaemontana divaricata* |
| Coronaridine | *Tabernaemontana divaricata* |
| Coronaridine hydroxyindole-nine | *Tabernaemontana divaricata* |
| Ervaticine | *Tabernaemontana divaricata* |
| Mehranine | *Tabernaemontana divaricata* |
| Erycristagallin | *Tamarix nilotica* |
| Myrcene | *Tamarix nilotica* |
| Pinocarvone | *Tamarix nilotica* |
| Tamarixetin | *Tamarix nilotica* |
| 3, 4-dihydroxhbenzoic acid | *Tanacetum Parthenium* |
| 3-methoxy-4-hydroxyienzoic acid | *Tanacetum Parthenium* |
| Adenosine | *Tanacetum Parthenium* |
| Alpha Tetrapathe | *Tanacetum Parthenium* |
| Apigenin | *Tanacetum Parthenium* |
| Benzaldehyde | *Tanacetum Parthenium* |
| Costinulide | *Tanacetum Parthenium* |
| Emodin | *Tanacetum Parthenium* |
| Narlumicine | *Tanacetum Parthenium* |
| Narlumidine | *Tanacetum Parthenium* |
| Oxysanguinarine | *Tanacetum Parthenium* |
| Sesquiterpene Glycoside | *Tanacetum Parthenium* |
| Xylan | *Tanacetum Parthenium* |
| 4a-Hydroxyskytanthine | *Tecoma stans* |
| 6-Hydroxydehydroskytanthine | *Tecoma stans* |
| Kinabalurine G | *Tecoma stans* |
| Noractinidine | *Tecoma stans* |
| Noroxyhydrastinine | *Thalictrum alpinum* |
| Thalibulamine | *Thalictrum cultratum* |
| Corunnine | *Thalictrum foetidum* |
| 2'-Noradiantifoline | *Thalictrum minus* |
| Adiantifoline | *Thalictrum minus* |
| Bursanine | *Thalictrum minus* |
| Dehydrothalmelatine | *Thalictrum minus* |
| Isothalisopavine | *Thalictrum minus* |
| Istanbulamine | *Thalictrum minus* |
| Iznikine | *Thalictrum minus* |
| O6'-Demethyladiantifoline | *Thalictrum minus* |
| Obaberine | *Thalictrum minus* |
| Ocoteine | *Thalictrum minus* |
| Thalactamine | *Thalictrum minus* |
| Thaliadanine | *Thalictrum minus* |
| Thalicmidine N-oxide | *Thalictrum minus* |
| Thalrugosine | *Thalictrum minus* |
| N-Methyldanguyelline | *Thalictrum pedunculatum* |
| Caffeine | *Theobroma cacao* |
| Clovamide | *Theobroma cacao* |
| Angoline | *Toddalia asiatica* |
| Arnottianamide | *Toddalia asiatica* |
| Cyclohexylamine | *Toddalia asiatica* |
| Dihydroavicine | *Toddalia asiatica* |
| Dihydrochelerythrine | *Toddalia asiatica* |
| Dihydrochelerythrine | *Toddalia asiatica* |
| Dihydronitidine | *Toddalia asiatica* |
| Norchelerythrine | *Toddalia asiatica* |
| Toddalidimerine | *Toddalia asiatica* |
| N1,N4-Diferuloylputrescine | *Tribulus terrestris* |
| Terrestriamide | *Tribulus terrestris* |
| Clovamide | *Trifolium pratense* |
| p-Coumaric acid | *Trifolium pratense* |
| Linamarin | *Trifolium repens* |
| Lotaustralin | *Trifolium repens* |
| Taxiphyllin | *Triglochin maritima* |
| Trigoxazonane | *Trigonella foenum-graecum* |
| N1-trans-Feruloylagmatine | *Triticum aestivum* |
| 14-Desoxy-13a-methyltylohirsutinidine | *Tylophora hirsuta* |
| Alihirsutine A | *Tylophora hirsuta* |
| Tyloindicine C | *Tylophora indica* |
| Dihydrocorynantheine | *Uncaria gambier* |
| Dihydrogambirtannine | *Uncaria gambier* |
| Gambirine | *Uncaria gambier* |
| Gambirtannine | *Uncaria gambier* |
| Neooxygambirtannine | *Uncaria gambier* |
| Oxogambirtannine | *Uncaria gambier* |
| 3-Acetyl-2,7-naphthyridine | *Valeriana officinalis* |
| Actinidine | *Valeriana officinalis* |
| Chatinine | *Valeriana officinalis* |
| N-(p-Hydroxyphenethyl)actinidine | *Valeriana officinalis* |
| 7-Methoxyflindersine | *Vepris bilocularis* |
| 7-Prenyloxyflindersine | *Vepris bilocularis* |
| NMethyl- 7-prenyloxyflindersine | *Vepris bilocularis* |
| Epinine | *Vicia faba* |
| N1,N4-Diferuloylputrescine | *Vicia faba* |
| N1,N4-Di-p-coumaroylputrescine | *Vicia faba* |
| N2-(2-Hydroxysuccinoyl)arginine | *Vicia faba* |
| N-Jasmonoyldopamine | *Vicia faba* |
| N-p-trans- Coumaroyltyramine | *Vicia faba* |
| Vicine | *Vicia faba* |
| Homohexamine | *Vicia sativa* |
| N,N'-Bis(4-aminobutyl)-1,4-butanediamine | *Vicia sativa* |
| N,N'-Bis(4-aminobutyl)-1,4-butanediamine, 9CI | *Vicia sativa* |
| N10-(4-Aminobutyl) homohexamine | *Vicia sativa* |
| N5-(4-Aminobutyl)- homohexamine | *Vicia sativa* |
| N5-(4-Aminobutyl)- homopentamine | *Vicia sativa* |
| N5-(4-Aminobutyl)-homospermine | *Vicia sativa* |
| N5,N10-Bis(4-aminobutyl)homopentamine | *Vicia sativa* |
| N5,N15-Bis(4-aminobutyl)homopentamine | *Vicia sativa* |
| Thermospermine | *Vicia sativa* |
| Vicine | *Vicia sativa* |
| Akuammine N-oxide | *Vinca major* |
| Dihydrovincarpine | *Vinca major* |
| Majorine | *Vinca major* |
| Majovine | *Vinca major* |
| Rauniticine | *Vinca major* |
| Serpentine | *Vinca major* |
| Vincarpine | *Vinca major* |
| Vinervinine | *Vinca major* |
| Propionylcholine | *Viscum album* |
| Anahygrine | *Withania somnifera* |
| 8-Acetonyldihydrochelerythrine | *Xylocarpus granatum* |
| 5-Ethoxychelerythrine | *Zanthoxylum nitidum* |
| 7,8-Dihydro-8-methoxynitidine | *Zanthoxylum nitidum* |
| 8-Methoxysanguinarine | *Zanthoxylum nitidum* |
| 9-Demethyl-8- methoxydihydrochelerythrine | *Zanthoxylum nitidum* |
| Dihydronitidine | *Zanthoxylum nitidum* |
| Fagaridine | *Zanthoxylum nitidum* |
| Norchelerythrine | *Zanthoxylum nitidum* |
| Oxychelerythrine | *Zanthoxylum nitidum* |
| Oxynitidine | *Zanthoxylum nitidum* |
| Oxyterihanine | *Zanthoxylum nitidum* |
| Terihanine | *Zanthoxylum nitidum* |
| Skimmianine | *Zanthoxylum ovalifolium* |
| Terihanine | *Zanthoxylum ovalifolium* |
| 7-Carboxyevodiamine | *Zanthoxylum rhetsa* |
| Pepuline | *Zanthoxylum rhetsa* |
| 4-Acetyl-2(3H)-benzoxazo-lone, 9CI | *Zea mays* |
| 4-Coumaroylputrescine | *Zea mays* |
| 6,7-Dimethoxy-1(3H)-benzoxazolone | *Zea mays* |
| Blepharin | *Zea mays* |
| Chaetoglobosin N | *Zea mays* |
| N1,N4-Diferuloylputrescine | *Zea mays* |
| Zeanoside B | *Zea mays* |
| Zeanoside C | *Zea mays* |
| Amphibine B | *Ziziphus oenoplia* |
| Amphibine A | *Zizyphus spinachristi* |
| Amphibine E | *Zizyphus spinachristi* |
| Amphibine F | *Zizyphus spinachristi* |
| Franganine | *Zizyphus spinachristi* |
| Mauritine C | *Zizyphus spinachristi* |

**Table S2:** ADMET of Screened 108 Phytochemicals

| **Phytochemicals** | **Plant Name** | **ESOL Class** | **GI absorption** | **BBB Penetration** | **Lipinski violations** | **Toxicity** | **Carcinogenicity** |
| --- | --- | --- | --- | --- | --- | --- | --- |
| (+)-medioresinol | *Aloe Vera* | Soluble | High | No | 0 | Non-Toxic | Non-Carcinogenic |
| (+)-syringaresinol | *Aloe Vera* | Soluble | High | No | 0 | Non-Toxic | Non-Carcinogenic |
| 12S-hydroxyandrographolide | *Fumaria Indica* | Soluble | High | No | 0 | Non-Toxic | Non-Carcinogenic |
| 19-hydroxy-8 (17), 13-labdadien-15, 16-olide | *Fumaria Indica* | Soluble | High | No | 0 | Non-Toxic | Non-Carcinogenic |
| 3, 4-dihydroxhbenzoic acid | *Tanacetum Parthenium* | Soluble | High | No | 0 | Non-Toxic | Non-Carcinogenic |
| 3-methoxy-4-hydroxyienzoic acid | *Tanacetum Parthenium* | Soluble | High | No | 0 | Non-Toxic | Non-Carcinogenic |
| 3-oxo-14-deoxy-andrographolide | *Fumaria Indica* | Soluble | High | No | 0 | Non-Toxic | Non-Carcinogenic |
| 5, 7, 3', 4'-tetrahydroxyflavone | *Solanum nigrum* | Soluble | High | No | 0 | Non-Toxic | Non-Carcinogenic |
| 5, 7, 4'-trihydroxyflavone | *Solanum nigrum* | Soluble | High | No | 0 | Non-Toxic | Non-Carcinogenic |
| AbyssinoneV | *Erythrina Varigatae* | Soluble | High | No | 0 | Non-Toxic | Non-Carcinogenic |
| Adenosine | *Tanacetum Parthenium* | Soluble | High | No | 0 | Non-Toxic | Non-Carcinogenic |
| Aloe Emodin | *Aloe Vera* | Soluble | High | No | 0 | Non-Toxic | Non-Carcinogenic |
| Alpha Tetrapathe | *Tanacetum Parthenium* | Soluble | High | No | 0 | Non-Toxic | Non-Carcinogenic |
| AmyrisinA | *Silybum Marianum* | Soluble | High | No | 0 | Non-Toxic | Non-Carcinogenic |
| AmyrisinB | *Silybum Marianum* | Soluble | High | No | 0 | Non-Toxic | Non-Carcinogenic |
| AmyrisinC | *Silybum Marianum* | Soluble | High | No | 0 | Non-Toxic | Non-Carcinogenic |
| AndrographidoidsA | *Santolina insularis* | Soluble | High | No | 0 | Non-Toxic | Non-Carcinogenic |
| Anthraxin | *Silybum Marianum* | Soluble | High | No | 0 | Non-Toxic | Non-Carcinogenic |
| Apigenin | *Tanacetum Parthenium* | Soluble | High | No | 0 | Non-Toxic | Non-Carcinogenic |
| ApigeninB | *Solanum nigrum* | Soluble | High | No | 0 | Non-Toxic | Non-Carcinogenic |
| Ascorbic Acid | *Fumaria Indica* | Soluble | High | No | 0 | Non-Toxic | Non-Carcinogenic |
| Benzaldehyde | *Tanacetum Parthenium* | Soluble | High | No | 0 | Non-Toxic | Non-Carcinogenic |
| Caffeic Acid | *Solanum nigrum* | Soluble | High | No | 0 | Non-Toxic | Non-Carcinogenic |
| Cannflavin | *Silybum Marianum* | Soluble | High | No | 0 | Non-Toxic | Non-Carcinogenic |
| Cirsimaritin | *Santolina insularis* | Soluble | High | No | 0 | Non-Toxic | Non-Carcinogenic |
| Costinulide | *Tanacetum Parthenium* | Soluble | High | No | 0 | Non-Toxic | Non-Carcinogenic |
| Derrisin | *Silybum Marianum* | Soluble | High | No | 0 | Non-Toxic | Non-Carcinogenic |
| Dihydroxy-348-trimethoxyxanthone | *Silybum Marianum* | Soluble | High | No | 0 | Non-Toxic | Non-Carcinogenic |
| Diprenyleriodictyol | *Silybum Marianum* | Soluble | High | No | 0 | Non-Toxic | Non-Carcinogenic |
| DoitunggarcinoneC | *Silybum Marianum* | Soluble | High | No | 0 | Non-Toxic | Non-Carcinogenic |
| Emodin | *Tanacetum Parthenium* | Soluble | High | No | 0 | Non-Toxic | Non-Carcinogenic |
| Epoxy | *Fumaria Indica* | Soluble | High | No | 0 | Non-Toxic | Non-Carcinogenic |
| Erycristagallin | *Tamarix nilotica* | Soluble | High | No | 0 | Non-Toxic | Non-Carcinogenic |
| Erythrinins B | *Erythrina Varigatae* | Soluble | High | No | 0 | Non-Toxic | Non-Carcinogenic |
| EryvarinM | *Erythrina Varigatae* | Soluble | High | No | 0 | Non-Toxic | Non-Carcinogenic |
| EryvarinO | *Erythrina Varigatae* | Soluble | High | No | 0 | Non-Toxic | Non-Carcinogenic |
| EryvarinolsA | *Erythrina Varigatae* | Soluble | High | No | 0 | Non-Toxic | Non-Carcinogenic |
| EryvarinP | *Erythrina Varigatae* | Soluble | High | No | 0 | Non-Toxic | Non-Carcinogenic |
| EryvarinQ | *Erythrina Varigatae* | Soluble | High | No | 0 | Non-Toxic | Non-Carcinogenic |
| EryvarinR | *Erythrina Varigatae* | Soluble | High | No | 0 | Non-Toxic | Non-Carcinogenic |
| Estafin | *Fumaria Indica* | Soluble | High | No | 0 | Non-Toxic | Non-Carcinogenic |
| EuchrenoneB | *Erythrina Varigatae* | Soluble | High | No | 0 | Non-Toxic | Non-Carcinogenic |
| Feruloyltyramine | *Fumaria Indica* | Soluble | High | No | 0 | Non-Toxic | Non-Carcinogenic |
| Fumaric Acid | *Solanum nigrum* | Soluble | High | No | 0 | Non-Toxic | Non-Carcinogenic |
| Fumaritine N-oxide | *Fumaria Indica* | Soluble | High | No | 0 | Non-Toxic | Non-Carcinogenic |
| Hydroxy Erythratidine | *Erythrina Varigatae* | Soluble | High | No | 0 | Non-Toxic | Non-Carcinogenic |
| Hydroxyanhydro | *Fumaria Indica* | Soluble | High | No | 0 | Non-Toxic | Non-Carcinogenic |
| Hydroxygenistein | *Erythrina Varigatae* | Soluble | High | No | 0 | Non-Toxic | Non-Carcinogenic |
| Hydroxymunduserone | *Silybum Marianum* | Soluble | High | No | 0 | Non-Toxic | Non-Carcinogenic |
| Isoach | *Fumaria Indica* | Soluble | High | No | 0 | Non-Toxic | Non-Carcinogenic |
| IsoerysenegalenseinE | *Erythrina Varigatae* | Soluble | High | No | 0 | Non-Toxic | Non-Carcinogenic |
| Isomangostin | *Silybum Marianum* | Soluble | High | No | 0 | Non-Toxic | Non-Carcinogenic |
| Isopomiferin | *Silybum Marianum* | Soluble | High | No | 0 | Non-Toxic | Non-Carcinogenic |
| IsosilybinA | *Silybum Marianum* | Soluble | High | No | 0 | Non-Toxic | Non-Carcinogenic |
| IsosilybinB | *Silybum Marianum* | Soluble | High | No | 0 | Non-Toxic | Non-Carcinogenic |
| Laburnetin | *Erythrina Varigatae* | Soluble | High | No | 0 | Non-Toxic | Non-Carcinogenic |
| Lenticin | *Erythrina Varigatae* | Soluble | High | No | 0 | Non-Toxic | Non-Carcinogenic |
| Lupiwighteone | *Erythrina Varigatae* | Soluble | High | No | 0 | Non-Toxic | Non-Carcinogenic |
| Luteolin | *Melissa officinalis* | Soluble | High | No | 0 | Non-Toxic | Non-Carcinogenic |
| Lycopene | *Moringa peregrina* | Soluble | High | No | 0 | Non-Toxic | Non-Carcinogenic |
| Mearnsetin | *Silybum Marianum* | Soluble | High | No | 0 | Non-Toxic | Non-Carcinogenic |
| Menisdaurin | *Andrographis paniculata* | Soluble | High | No | 0 | Non-Toxic | Non-Carcinogenic |
| Mundulinol | *Silybum Marianum* | Soluble | High | No | 0 | Non-Toxic | Non-Carcinogenic |
| Myrcene | *Tamarix nilotica* | Soluble | High | No | 0 | Non-Toxic | Non-Carcinogenic |
| Narlumicine | *Tanacetum Parthenium* | Soluble | High | No | 0 | Non-Toxic | Non-Carcinogenic |
| Narlumidine | *Tanacetum Parthenium* | Soluble | High | No | 0 | Non-Toxic | Non-Carcinogenic |
| Noroxyhydrastinine | *Andrographis paniculata* | Soluble | High | No | 0 | Non-Toxic | Non-Carcinogenic |
| OphiopogonanoneG | *Silybum Marianum* | Soluble | High | No | 0 | Non-Toxic | Non-Carcinogenic |
| Osajin | *Erythrina Varigatae* | Soluble | High | No | 0 | Non-Toxic | Non-Carcinogenic |
| Oxyresveratrol | *Erythrina Varigatae* | Soluble | High | No | 0 | Non-Toxic | Non-Carcinogenic |
| Oxysanguinarine | *Tanacetum Parthenium* | Soluble | High | No | 0 | Non-Toxic | Non-Carcinogenic |
| Pantothenic Acid | *Fumaria Indica* | Soluble | High | No | 0 | Non-Toxic | Non-Carcinogenic |
| Papracinine | *Fumaria Indica* | Soluble | High | No | 0 | Non-Toxic | Non-Carcinogenic |
| Paprafumine | *Fumaria Indica* | Soluble | High | No | 0 | Non-Toxic | Non-Carcinogenic |
| Papraine | *Fumaria Indica* | Soluble | High | No | 0 | Non-Toxic | Non-Carcinogenic |
| Papraline | *Santolina insularis* | Soluble | High | No | 0 | Non-Toxic | Non-Carcinogenic |
| Paprarine | *Fumaria Indica* | Soluble | High | No | 0 | Non-Toxic | Non-Carcinogenic |
| Parthenolide | *Santolina insularis* | Soluble | High | No | 0 | Non-Toxic | Non-Carcinogenic |
| Pinocarvone | *Tamarix nilotica* | Soluble | High | No | 0 | Non-Toxic | Non-Carcinogenic |
| Potassium Sorbate | *Solanum nigrum* | Soluble | High | No | 0 | Non-Toxic | Non-Carcinogenic |
| Pyridoxine | *Santolina insularis* | Soluble | High | No | 0 | Non-Toxic | Non-Carcinogenic |
| Raddeanine | *Andrographis paniculata* | Soluble | High | No | 0 | Non-Toxic | Non-Carcinogenic |
| Rhamnetin | *Solanum nigrum* | Soluble | High | No | 0 | Non-Toxic | Non-Carcinogenic |
| Riboflavin | *Andrographis paniculata* | Soluble | High | No | 0 | Non-Toxic | Non-Carcinogenic |
| Robustone | *Erythrina Varigatae* | Soluble | High | No | 0 | Non-Toxic | Non-Carcinogenic |
| SchizolaenoneB | *Silybum Marianum* | Soluble | High | No | 0 | Non-Toxic | Non-Carcinogenic |
| Sesquiterpene Glycoside | *Tanacetum Parthenium* | Soluble | High | No | 0 | Non-Toxic | Non-Carcinogenic |
| SigmoidinA | *Erythrina Varigatae* | Soluble | High | No | 0 | Non-Toxic | Non-Carcinogenic |
| SigmoidinB | *Erythrina Varigatae* | Soluble | High | No | 0 | Non-Toxic | Non-Carcinogenic |
| SigmoidinC | *Erythrina Varigatae* | Soluble | High | No | 0 | Non-Toxic | Non-Carcinogenic |
| SilybinA | *Silybum Marianum* | Soluble | High | No | 0 | Non-Toxic | Non-Carcinogenic |
| SilybinB | *Silybum Marianum* | Soluble | High | No | 0 | Non-Toxic | Non-Carcinogenic |
| SilybinC | *Silybum Marianum* | Soluble | High | No | 0 | Non-Toxic | Non-Carcinogenic |
| SilybinD | *Silybum Marianum* | Soluble | High | No | 0 | Non-Toxic | Non-Carcinogenic |
| Silydianin | *Silybum Marianum* | Soluble | High | No | 0 | Non-Toxic | Non-Carcinogenic |
| silydianinB | *Silybum Marianum* | Soluble | High | No | 0 | Non-Toxic | Non-Carcinogenic |
| Stachydrine | *Erythrina Varigatae* | Soluble | High | No | 0 | Non-Toxic | Non-Carcinogenic |
| Tamarixetin | *Tamarix nilotica* | Soluble | High | No | 0 | Non-Toxic | Non-Carcinogenic |
| Tanaparthe | *Solanum nigrum* | Soluble | High | No | 0 | Non-Toxic | Non-Carcinogenic |
| Tanaparthe | *Solanum nigrum* | Soluble | High | No | 0 | Non-Toxic | Non-Carcinogenic |
| Tanetin | *Santolina insularis* | Soluble | High | No | 0 | Non-Toxic | Non-Carcinogenic |
| TanetinB | *Silybum Marianum* | Soluble | High | No | 0 | Non-Toxic | Non-Carcinogenic |
| Taxifolin | *Silybum Marianum* | Soluble | High | No | 0 | Non-Toxic | Non-Carcinogenic |
| Thiamine | *Andrographis paniculata* | Soluble | High | No | 0 | Non-Toxic | Non-Carcinogenic |
| TomentodiplaconeB | *Silybum Marianum* | Soluble | High | No | 0 | Non-Toxic | Non-Carcinogenic |
| Uric Acid | *Andrographis paniculata* | Soluble | High | No | 0 | Non-Toxic | Non-Carcinogenic |
| Wighteone | *Erythrina Varigatae* | Soluble | High | No | 0 | Non-Toxic | Non-Carcinogenic |
| Xylan | *Tanacetum Parthenium* | Soluble | High | No | 0 | Non-Toxic | Non-Carcinogenic |
